# *Cox*1 barcoding *versus* multilocus species delimitation: validation of two mite species with contrasting effective population sizes

**DOI:** 10.1186/s13071-018-3242-5

**Published:** 2019-01-05

**Authors:** Pavel B. Klimov, Maciej Skoracki, Andre V. Bochkov

**Affiliations:** 1 Department of Ecology and Evolutionary Biology, Museum of Zoology, University of Michigan, 3600 Varsity Drive, Ann Arbor, Michigan 48108 USA; 2 Tyumen State University, 10 Semakova Str, 625003 Tyumen, Russia; 3 Department of Animal Morphology, Faculty of Biology, Adam Mickiewicz University, Umultowska 89, 60-614 Poznan, Poland; 4 Zoological Institute, Russian Academy of Sciences, Universitetskaya emb. 1, 199034 St Petersburg, Russia

**Keywords:** Species delimitation, *cox*1, Barcoding, Large population size, Mito-nuclear discordance

## Abstract

**Background:**

The *cox*1-barcoding approach is currently extensively used for high-throughput species delimitation and discovery. However, this method has several limitations, particularly when organisms have large effective population sizes. Paradoxically, most common, abundant, and widely distributed species may be misclassified by this technique.

**Results:**

We conducted species delimitation analyses for two host-specific lineages of scab mites of the genus *Caparinia*, having small population sizes. *Cox*1 divergence between these lineages was high (7.4–7.8%) while that of nuclear genes was low (0.06–0.53%). This system was contrasted with the medically important American house dust mite, *Dermatophagoides farinae*, a globally distributed species with very large population size. This species has two distinct, sympatric *cox*1 lineages with 4.2% divergence. We tested several species delimitation algorithms PTP, GMYC, ABGD, BPP, STACEY and PHRAPL, which inferred different species boundaries for these entities. Notably, STACEY recovered the *Caparinia* lineages as two species and *D. farinae* as a single species. BPP agreed with these results when the prior on ancestral effective population sizes was set to expected values, although delimitation of *Caparinia* was still equivocal. No other *cox*1 species delimitation algorithms inferred *D. farinae* as a single species, despite the fact that the nuclear *CPW2* gene shows some evidence for introgression between the *cox*1 groups. This indicates that the *cox*1-barcoding approach may result in excessive species splitting.

**Conclusions:**

Our research highlights the importance of using nuclear genes and demographic characteristics to infer species boundaries rather than relying on a single-gene barcoding approach, particularly for putative species having large effective population sizes.

**Electronic supplementary material:**

The online version of this article (10.1186/s13071-018-3242-5) contains supplementary material, which is available to authorized users.

## Background

The DNA barcoding approach is a useful tool for DNA-based, automatic identification of organisms. Because this approach relies on sequencing of a standardized gene region, the “barcode”, a specimen can be identified by comparing its sequence to a reference database [1, 2], for example, GenBank or BOLD [3]. Typically, for animals, the standard locus is the Folmer fragment of the mitochondrial gene, cytochrome *c* oxidase subunit 1 (*cox*1) [2], for fungi it is ITS2 [4], while for plants, two loci from the plastid genome are used [5]. To be successful, a DNA barcoding approach should meet three basic criteria: (i) a sufficient amount of variation exists in the barcode region to distinguish species; (ii) no overlap between intra- and inter-specific genetic distances; and (iii) a prior knowledge of species boundaries. Here, the notion of a barcoding gap, a “break” in the distribution among within- and between-species variation distances, is very important. In practice barcoding gap analyses are widely used for species delimitation, assigning specimens to species when species boundaries are unknown, often in conjunction with building a phylogenetic or distance-based tree [6, 7]. In many cases, no single threshold or barcoding gap exist that can be used to assign all specimens without incurring high error rates [7–10]. Typical barcoding gap values (Kimura 2-parameter genetic distances, K2P) range between ~2 to 4%, above which genetic distances are considered to be interspecific [3, 6, 10–13]. These values can be either used as predetermined thresholds [11] or, more appropriately, as useful prior threshold values in automatic gap discovery analyses [14]. However, some species, particularly those having large population sizes, show maximum within-species *cox*1 distances much higher than these values: 15.4% in the Chinese perch *Siniperca chuatsi* [10]; 10.1% in the human follicle mite *Demodex folliculorum* (Demodecidae) (conservatively recalculated from [15]); 5.7–6.8% in the common blue butterfly *Polyommatus icarus* (Lycaenidae) [16]; about 6% in the sea snail *Echinolittorina vidua* (Littorinidae) [17]; 4.3% in the mold mite *Tyrophagus putrescentiae* [18]; and 4.2% in the American house dust mite (our data) to name a few. *Cox*1 barcoding performs well when species have small population sizes, low speciation rates [19] or substantial divergence times [10]. Thus, paradoxically, most common and widely distributed species, such as those listed above, are in the ‘gray zone’ of the *cox*1 barcoding approach and may present methodological challenges for the DNA barcoding approach.

Population genetic theory-based alternatives to threshold-based approaches can accurately delimit species under a range of conditions, including variable population sizes and times of origins [8, 20]. Two recently proposed species delimitation methods, BPP [21] and STACEY [22], are both based on the multispecies coalescent model and assume that species are distinct populations without gene flow. The latter is estimated by taking into account the ancestral population size and time of divergence at the root, while species trees are estimated under a coalescent process, assuming neutral evolution and no selection for single or multiple loci. When all these parameters are estimated (or fixed to a known value), posterior probabilities for alternative species delimitation models can be calculated, and the best-fitting model can be selected objectively. Another species delimitation approach that uses multispecies coalescent, PHRAPL [23], is based on a likelihood framework and, in addition, also incorporates gene flow when estimating species boundaries. The disadvantages of these methods are: (i) the need to estimate population genetic parameters that are typically unknown (except for PHRAPL, which estimates them using Maximum Likelihood); (ii) use of phased sequences of nuclear loci (i.e. polymorphisms in sequences should be phased out to represent the two alleles of a diploid organism); (iii) *a priori* specimen assignment to a ‘minimal’ population in several cases; and (iv) the assumption of neutral evolution. In addition, multispecies coalescent methods can be computationally prohibitive and are only feasible for small sets of species with unclear boundaries. Despite being methodologically superior, multispecies coalescent methods have their own ‘gray zone’ where conflicting species delimitations are possible - typically, when gene trees have shallow branch lengths (recent speciation events) and when lineages have small effective population sizes (higher probability of speciation due to drift).

Here we explore several methods of species delimitations, the threshold-based ABGD [14], the multispecies coalescent-based BPP, STACEY and PHRAPL, as well as other algorithms, GMYC [24] and PTP [25]. Our specific goal was to evaluate the species status of mostly host-specific populations of scab mites of the genus *Caparinia* (family Psoroptidae) parasitizing two species of hedgehogs, the European hedgehog *Erinaceus europaeus* and the African hedgehog *Atelerix albiventris* [26–30]; the latter species being a popular pet throughout the world. K2P *cox*1 distances between the two populations were 7.48–7.77% (our data). These mites are rare in the field (our data; Additional file 1: Text S1), suggesting that their population sizes are relatively small. Despite the large *cox*1 distances between these populations, nuclear genes of these lineages show only minimal variation (0.09–0.53%; our data, see below). Phenotypic differences were also minimal and do not allow clear-cut taxonomic judgment on whether these populations are either a single or separate species [31, 32]. Therefore, our model system allows testing whether distinct *cox*1-based clades are sufficient to delimit species when nuclear genes form shallow clades and phenotypic differences between lineages are minimal, which might suggest a recent divergence event between these lineages and, therefore, rapid speciation rates. Thus, our empirical system may be in the ‘gray zone’ of molecular taxonomy. For comparative purposes, we also employ another model system, the American house dust mite *Dermatophagoides farinae*, which is a globally distributed species with a large population size. It has a strongly structured population with two *cox*1 lineages having a 4.19% K2P divergence. To calculate a barcoding gap without potential influence of technical errors or removing the 5% “outliers” [9, 33], we employ a well-curated *cox*1 sequence database (Additional file 2: Table S1), including two closely related families, the psoroptic scab mites (Psoroptidae) and pyroglyphid house dust mites (Pyroglyphidae). These families contain cosmopolitan, free-living species with large effective population sizes (house dust mites *Dermatophagoides farinae* and *D. pteronyssinus*), and either multiple- (*Psoroptes ovis*, *Chorioptes bovis*) or single-host (*Choirioptes sweatmani*) parasites.

## Results

### Quality of GenBank data

Out of 12 pyroglyphid *cox*1 GenBank sequences (Additional file 3: Figure S1), 10 (83.3%) were excluded: *Dermatophagoides farinae* China (KP871846.1-KP871850.1, KX211988.1-KX211990.1; unusual amino acid substitutions); *Dermatophagoides pteronyssinus* Thailand (HQ823623.1; unusual amino acid substitutions, stop codons, and frameshifting insertions); *Dermatophagoides farinae* Thailand (HQ823622.1; unusual amino acid substitutions, stop codons, and frameshifting insertions). Only two sequences (16.7%) passed our quality filter criterion: *Dermatophagoides pteronyssinus* Belgium (EU884425.1) and *Euroglyphus maynei* USA (MUJZ01072749.1; annotated alignment in Additional file 4). Low quality sequences tend to occupy basal positions within species subclades, e.g. groups 1 and 2 of *Dermatophagoides farinae*, creating a false impression of their earlier origins (Additional file 3: Figure S1). After removal of the suspect sequences, minimun-maximum K2P *cox*1 genetic distances changed only marginally: *Dermatophagoides microceras vs D. farinae* (9.34–10.02 *vs* 9.00–10.22% before the removal); *D. farinae vs D.farinae* (maximum of 4.19 *vs* 4.57% before the removal); *D. pteronyssinus vs D. pteronyssinus* (maximum of 1.97 *vs* 2.14% before the removal).

### Morphological differences

**Fig. 1:**
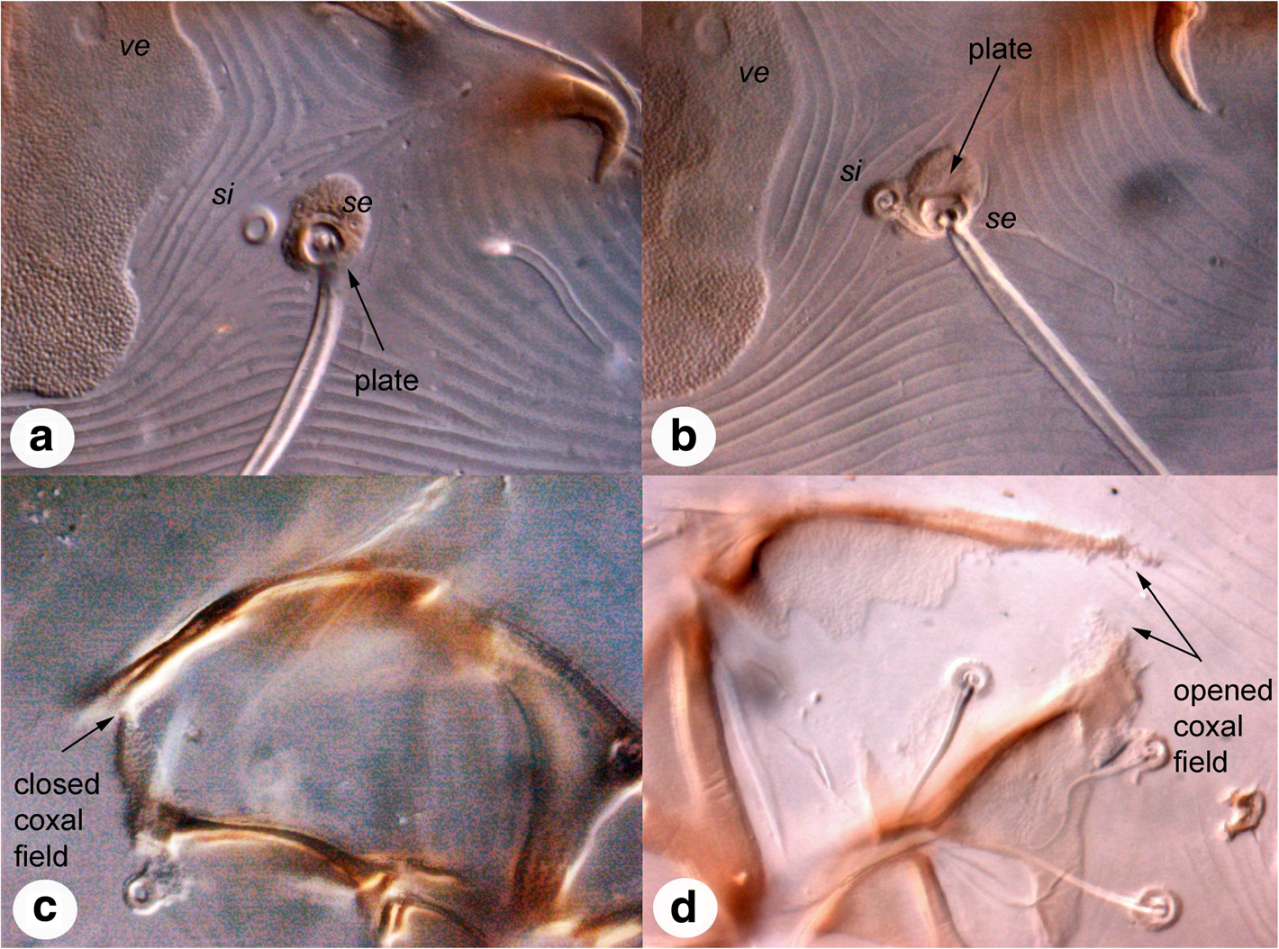
Diagnostic characters of *Caparinia tripilis* and *C. ictonyctis*. **a** Position of seta *si*, female of *C. ictonyctis* stat. res. **b** Position of seta *si*, female of *C. tripilis*; **c** Coxal field III, male of *C. ictonyctis* stat. res. **d** Coxal field III, male of *C. tripilis*

We found the following differences between *Caparinia tripilis versus* mites from *Atelerix albiventris* and *Ictonyx striatus* (hereafter referred to as *Caparinia ictonyctis*, see the Discussion section). In females of *C. ictonyctis*, setae *si* are situated off the small plates bearing setae *se* (Fig. 1a), while in *C. tripilis* these setae are on or, more rarely, off, the small plates (Fig. 1b). In males of *C. ictonyctis*, coxal fields III are completely closed (Fig. 1c), while in *C. tripilis*, coxal fields III are semienclosed (Fig. 1d).

### Genetic distances

To calculate a barcoding gap without potential influence of technical errors or removing the 5% “outliers”, we employed a well-curated *cox*1 sequence database, including two closely related families, the psoroptic scab mites (Psoroptidae) and pyroglyphid house dust mites (Pyroglyphidae).

**Fig. 2:**
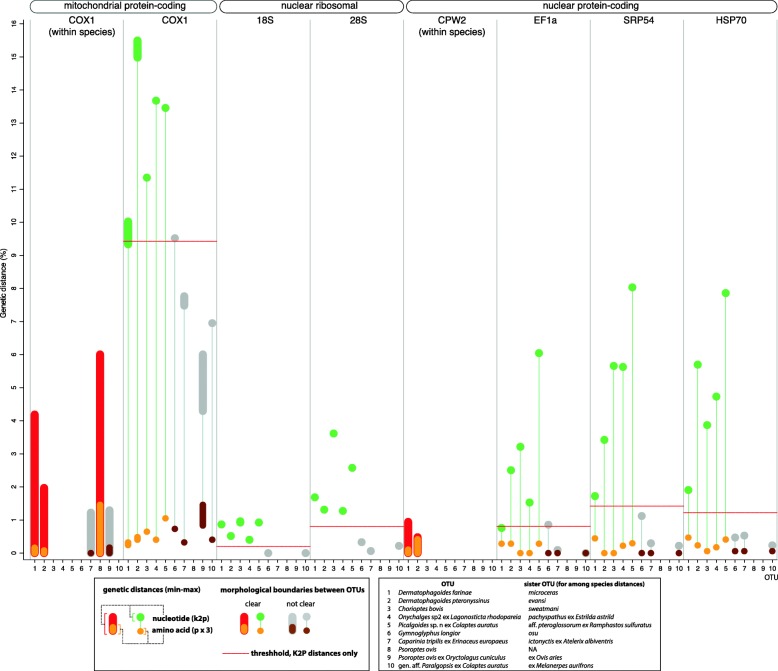
Comparison within- and among-species max-min genetic distances across mitochondrial protein-coding, nuclear ribosomal, and nuclear protein-coding genes (a total of 7 loci). Nucleotide distances (K2P) are contrasted with uncorrected amino acid distances (multiplied by 3 to be in the same scale). Nucleotide distances of putative species (OTUs) having no clear morphological boundaries are shown in gray, while OTUs with clear morphological species are shown in red (within-species) or green (among-species distances). Threshold in nucleotide K2P distances between these two groups of OTUs is shown by a horizontal line. Within-species distances are given only for the three taxa where sufficient numbers of individuals were sequenced and genetic variation was detected: *Dermatophagoides farinae*, *D. pteronyssinus* and *Psoroptis ovis*

Among the seven loci, the mitochondrial protein-coding gene *cox*1 had the largest within- and among-species distances (0–6.0% and 4.3–15.5%, respectively) (Fig. 2, Additional file 5: Table S2). Nuclear genes with the highest between-species K2P distances were *SRP54* (0.2–8.0%) and *HSP70* (0.2–7.9%), while *18S* had the lowest genetic distances (0–1.0%) (Fig. 2, Additional file 5: Table S2). For nuclear genes, within-species distances were available only for *CPW2*: 0–0.95% (*Dermatophagoides farinae*) and 0–0.48% (*D. pteronyssinus*) (Additional file 6: Figure S2: contract of *cox*1 *vs CPW2* phylogenies).

There was no clear threshold between within- and between-species *cox*1 distances, given the fact that putative species with no clear morphological differences may be or may not be true species (Fig. 2; shown by gray) or may represent two or more true species (e.g. *Psoroptis ovis*). Nevertheless, for *cox*1, a ‘conservative’ threshold of > 9.52%, e.g. 9.6–10% in K2P distances, could distinguish all ‘good’ species, i.e. those having clear morphological differences (Fig. 2).

If the extreme value of *CPW2* within-species distances (0.95%) is taken as an ‘universal’ species cut-off for other nuclear genes, then misclassifications will occur for OTUs with no clear morphological differences for all genes (Table 1; compare 0.95% with minimum values; Fig. 2). For OTUs with clear morphological differences, misclassifications will occur in two loci, *EF1-α* and *18S*, which have minimum between-species distances below this threshold (Table 1, Fig. 2). It is notable, that in *D. pteronyssinus*, *CPW2* is probably under a strong selection because the ratio of synonymous *vs* non-synonymous mutations is very high (Fig. 2).

**Table 1:** Comparison of genetic distances (K2P) between two groups of putative species: with and without clear morphological differences

Locus	Morphological differences between OTUs					
	Clear		Not clear			
	Min	Max	Min	Max	Gap	Threshold
*cox*1	9.3369	15.4977	4.2953	9.5194	-0.1825	9.4281 (9.5194)
*SRP54*	1.7246	8.0358	0.2226	1.1215	0.6031	1.4230
*HSP70*	1.9088	7.8612	0.2352	0.5301	1.3787	1.2194
*EF1-α*	0.7607	6.0463	0.0000	0.8577	-0.0969	0.8092 (0.8577)
*28S*	1.2775	3.6173	0.0632	0.3302	0.9473	0.8039
*18S*	0.4043	0.9641	0.0000	0.0000	0.4043	0.2022

*Abbreviations*: Min, minimum; Max, maximum

Even though it was not possible to establish a universal species delimitation gap for nuclear genes, most loci (*SPR54*, *HSP70*, *28S*, *18S*) have a clear K2P gap between putative species with and without clear morphological differences (Table 1, Fig. 2), although distances for *cox*1 and *EF1-α* slightly overlapped (Table 1, Fig. 2).

Amino acid distances lack a clear threshold-like pattern allowing distinguishing either among putative or ‘good’ species (Additional file 5: Table S2, Fig. 2). For example, ‘good’ species *Chorioptes bovis* and *Ch. sweatmani* lack any amino acid substitutions for *EF1-α* and *SPR54*, while *HSP70* had only a single substitution.

### Species delimitation

#### GMYC

Analyses using trees inferred under different speciation models (i.e. Yule *vs* coalescent) and molecular evolution (i.e. relaxed *vs* strict clock) resulted in the same species delimitation scheme containing 49 species and nearly the same threshold times, -0.0131 to -0.0126 (Additional file 7: Table S3: columns 5–6). This scheme was exactly the same as the one found by the PTP Maximum Likelihood and ABGD (X1 = 1.1, P = 1.29%) analysis (see below).

#### PTP

The Maximum Likelihood solution had 49 species, which was exactly the same found by GMYC (see above) and ABGD with X = 1.1 (see below), where *Caparinia*, *Dermatophagoides farinae* and *Psoroptes ovis* were each split into two separate species (Additional file 7: Table S3: columns 1–2). The Bayesian solution had 52 species; the difference was due to excessive oversplitting of *Psoroptes ovis* ex *Ovis aries* and *Dermatophagoides farinae* group 1 (Additional file 7: Table S3: columns 3–4).

#### ABGD

**Fig. 3:**
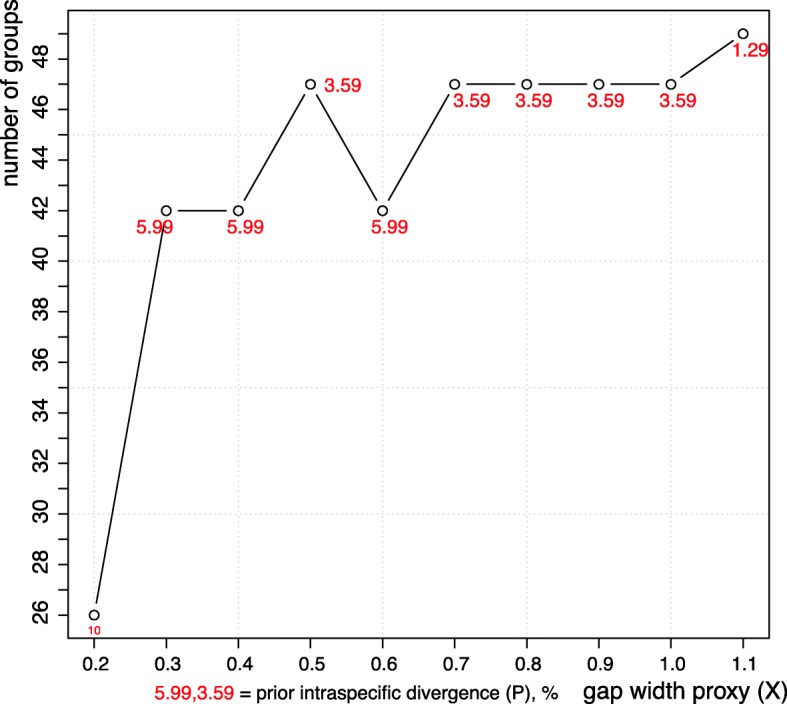
Summary of 10 ABGD runs with the gap width proxy parameter varied in the range from 0.2 to 1.1. Number of groups (putative species) recovered by the last multispecies ABGD iteration as well as the barcoding gap value are reported

The highest possible value of the barcoding gap width proxy parameter (X = 1.1) gave a 49-species delimitation (Fig. 3), exactly the same as the PTP Maximum Likelihood and GMYC solutions (Additional file 7: Table S3: columns 7–8). A range of lower values (X = 1.0–0.7 and 0.5) resulted in a 47-species scenario where *D. farinae* and *Psoroptes ovis* were each a single species, but the two *Caparinia* OTUs were still two separate species (Additional file 7: Table S3: columns 9–10). Lower values of X (X = 0.6 and 0.4) yielded a 42-species delimitation (Additional file 7: Table S3: columns 11–12). Notably, all “gray zone” taxon pairs (weak or no morphological differences) were collapsed (Fig. 2, Additional file 7: Table S3: columns 7–12). In addition, *Microlichus* sp. ex *Hirundo rustica* (Russia) and *Microlichus* sp. ex *Amazilia tzacatl* (Mexico) were collapsed to a single species; and *Dermatophagoides microceras* was collapsed with *Dermatophagoides farinae* (closely related species having distinct shapes of the female spermatheca). Setting the barcoding gap width proxy to X = 0.2 resulted in a 26-species delimitation scheme (Fig. 3). Many well-recognized species from different genera or families were collapsed to a single one. For example, *Picalgoides* spp., *Mesalgoides* spp., *Paralgopsis* spp. and *Onychalges* spp. were recovered as a single species (Additional file 7: Table S3: columns 13–14). Because of a major decrease of sensitivity of the method with X = 0.2, no further analyses were performed. Prior intraspecific divergence was strictly negatively correlated with the number species recovered (Fig. 3): 1.29% = 49 species; 3.59% = 47 species; 5.99% = 42 species; and 10% = 26 species (Fig. 3). Notice that these values represent a *prior* intraspecific divergence, which is used by the program to find a barcoding gap *above* the given value.

#### BPP

For the *Caparinia* dataset, analyses with the three sets of priors, reflecting different ancestral population sizes (θ) and root ages (τ0), all inferred a two-species model, lumping *Caparinia ictonyctis* from *Atelerix albiventris* together with *C. tripilis* from *Erinaceus europaeus* into a single species (Table 2). Posterior support for this model was moderate (0.863, 0.787), or low (0.514) for the model assuming both small population sizes at root and root age (Table 2). All analyses suggest a large decrease, 90.13–93.20%, of effective population size at the divergence of the two *Caparinia* OTUs (Table 2). For the *Dermatophagoides* dataset, analyses using the three sets of population genetics priors differed in whether *Dermatophagoides farinae* OTUs, Dfa and DFb, are a single or two separate species. When ancestral population size (θ) and root ages (τ) are large then these two OTUs are recovered as a single species with high probability [PP = 0.9537 (model), PP = 0.9886 (species)], while analyses with other priors suggest that these two mitochondrial-only groupings are separate species, with weak support for the 4-OTU species delimitation model + topology (PP = 0.5428, 0.5917; Table 2). However, posterior probabilities for the two OTUs (DFa, DFb) being separate species were high, 1.0–0.9585 and 0.9990–0.9480, respectively (Table 2).

**Table 2:** Summary of BPP species delimitation analyses of *Caparinia* (5 loci) and *Dermatophagoides* (2 loci) datasets using three sets of priors for ancestral population size (θ) and root age (τ0). Parameter estimates (means, 2.5-97.5% HPD intervals), posterior probabilities (PP) for select species delimitation models and OTUs are given

Species	Species tree/Pr	Prior distributions					
		θ ~ G(1, 10) τ0 ~ G(1, 10)		θ ~ G(2, 1000) τ0 ~ G(2, 1000)		θ ~ G(1, 10) τ0 ~ G(2, 1000)	
		PP/mean	Sampled HPD	PP/mean	Sampled HPD	PP/mean	Sampled HPD
	*Caparinia*						
	θ(root)	0.1851	0.0776–0.3245	0.0304	0.0229–0.0383	0.1867	0.0775–0.3227
	θ(Caic,Catri)	0.0167	0.0004–0.0437	0.003	0.0004–0.0061	0.0127	0.0004–0.0343
	τ(root)	0.0037	0.0002–0.0075	0.0015	0.0002–0.0031	0.0024	0.0003–0.0043
	τ(Caic,Catri)	0.0003	0–0.0008	0.0005	0–0.001	0.0003	0–0.0008
2	(Ocy, CaicCatri)	0.86285	86,285	0.51417	51,417	0.78684	78,684
3	(Ocy, (Caic, Catri))	0.13035	13,035	0.48054	48,054	0.21061	21,061
1	(OcyCaicCatri)	0.0068	680	na	na	0.00237	237
	Pr(Ocy)	0.9932	99,320	0.999	99,900	0.99763	99,763
	Pr(CaicCatri)	0.86285	86,285	0.51417	51,417	0.78684	78,684
	Pr(Caic)	0.13035	13,035	0.48531	48,531	0.21079	21,079
	Pr(Catri)	0.13035	13,035	0.48535	48,535	0.21079	21,079
	Pr(OcyCaicCatri)	0.0068	680	na	na	0.00237	237
	*Dermatophagoides*						
	θ(root)	0.2105	0.0193–0.4115	0.0341	0.0262–0.0425	0.2297	0.095–0.3978
	θ(DFa,DFb)	0.0407	0.0051–0.0954	0.0037	0.0016–0.0069	0.0048	0.0021–0.0087
	τ(root)	0.0141	0.0029–0.0335	0.0050	0.0018–0.0085	0.0107	0.0009–0.0258
	τ(DFa,DFb)	0.0004	0–0.001	0.0003	0–0.0008	0.0003	0–0.0008
4	(DP,(DM,(DFa,DFb)))	0.0104	1042	0.5428	54,284	0.5917	59,174
3	(DP,(DM,DFaDFb))	0.9537	95,373	na	na	0.0266	2661
	Pr(DFa)	0.0115	1145	1.0000	100000	0.9585	95,845
	Pr(DFb)	0.0115	1145	0.9990	99903	0.9480	94,802
	Pr(DFaDFb)	0.9886	98,855	na	na	0.0416	4155

*Abbreviation*: na, not available

#### STACEY

For the *Caparinia* dataset, the model treating the two host-specific *Caparinia* lineages as different species had a better relative fit than the model treating these lineages as a single species. Marginal likelihoods for these models were -16156.3 ± 0.173 *vs* -16161.8 ± 0.161, respectively (mean ± SE). The difference was BF = 5.56, suggesting that there is positive evidence for the two *Caparinia* species: *C. tripilis* and *C. ictonyctis*. For the *Dermatophagoides* dataset, an analysis where the two groups of *Dermatophagoides farinae* (Dfa and DFb) were merged into a single species (“minimal cluster”) had a better relative fit than the species delimitation model treating these two groups as two distinct species. Marginal likelihoods for these models were (mean ± SE): -5932.4 ± 0.14 *vs* -5935.5 ± 0.36, respectively. The difference was BF = 3.01, suggesting that there is positive evidence for the model treating *Dermatophagoides farinae* as a single species. Similarly, a STACEY species discovery analysis grouped the two *D. farinae* groups into a single species (Additional file 8: Figure S3).

#### PHRAPL

For *Dermatophagoides farinae*, among the nine PHRAPL models with ΔAIC less than 2, all were 3- and 2-species models (Additional file 9: Table S1). The best model (AIC 54.53) was a 3-species, isolation-only model (no gene flow), the second best model (AIC 55.47) was a 3-species, isolation + migration model, with two symmetrical migration rates: clades 1 and ancestral clade 2 + 3, and clades 2 and 3. The third best-scoring model (AIC 55.49) was a 2-species, isolation-only model, where clades 2 and 3 were collapsed. In all these models, gdi scores for clade 1 + ancestror for clades 2 + 3 (i.e. basal dichotomy of *Dermatophagoides farinae*) were high (0.994, 0.999 and 0.995, respectively); while gdi scores for clades 2+3 were medium or high (0.524, 0.953 and 0.524, respectively). The best-fitting 1-species model was a migration-only model (dAIC = 6.83, gdi = 0.001).

## Discussion

### Morphological discontinuities, genetic distances, and species delimitation

Even though using predetermined thresholds for species delimitation quickly falls into disrepute, the knowledge of approximate values separating within- *versus* between species genetic distances is still important. For example, it can be used to filter out suspect sequences (misidentifications, sequencing artifacts) from public databases [9, 33] or as a starting point (prior) in automatic gap discovery analyses [14]. Misspecification of this prior may result in inaccuracies in species delimitation by this method. Based on our curated Pyroglyphidae + Psoroptidae dataset, a ‘conservative’ distance of > 9.52% K2P distance was able to distinguish species that have clear morphological differences (Table 1). This value is very close to the average smallest interspecific distances (9%) reported for feather mites [34]. Below the 9.52% ‘conservative’ distance there was a “gray” species delimitation zone, where OTUs could not be unambiguously assigned to species based on morphology. It is notable that our ‘conservative’ *cox*1 threshold is much higher than values used in literature (4% [11], 3.14% [34], 3% [6, 12], ~2% [7, 12, 13], or lower [6]). Applying even the highest of these threshold values to our dataset will split species having large, strongly structured and presumably panmictic populations. For example, in the American house dust mite, *Dermatophagoides farinae*, *cox*1 suggests the existence of two distinct groups, 1 and 2 (Additional file 6: Figure S2) having a maximum K2P distance of 4.2%. However, the nuclear *CPW2* gene did not support these *cox*1-only groupings (Additional file 6: Figure S2), suggesting that, while some population structure does exist, members of different lineages are likely to interbreed (as evidenced by *CPW2* polymorphic individuals), and there is gene flow between them. Alternative explanation for this pattern is very recent lineage divergence. Similarly, *Psoroptes ovis*, a parasitic scab mite known from a wide range of domesticated and wild animals, forms two sister groups clearly separated by the nuclear *ITS* locus and microsatellites [35–38]. These groups are not host-specific and do not have clear morphological differences [36, 39]; one of them, the minority group, probably corresponds to our ‘rabbit’ group (*cox*1 K2P = 6.0%). Given our results, we believe that OTUs delimited by *cox*1 genetic distances lower than 9.52% need to be corroborated by independent lines of evidence, such as sequences of nuclear genes or breeding experiments for sexual species, rather than taken as conclusive evidence for the presence of distinct species. In contrast to *cox*1, nuclear genes showed variable thresholds from 0.2 to 1.4%, with *SPR54* and *HSP70* thresholds being the highest, and *18S* being the lowest (Table 1).

### *cox*1 barcode species delimitation

**Fig. 4:**
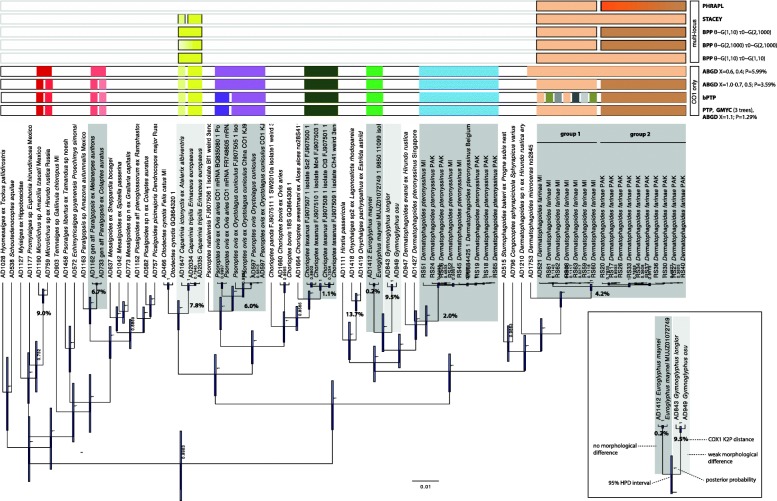
Summary of species delimitation analyses mapped on a 7-locus phylogenetic tree inferred in BEAST v2.4.7. The partitioning scheme contained 4 partitions and 7 loci: 18S stem+28 stem (substitution model: TVM+I+G), 18S loop+28S loop (TIM+I+G), EF1-α+SRP54+HSP70+CPW2 (GTR+I+G), *cox*1 (GTR+I+G). Additional file 10 provides the nexus datamatrix used to generate the 7-locus tree

There was a total of 42–49 plausible species delimitation schemes based on *cox*1; two analyses resulted in an abnormally high (54, bPTP) or low (26, ABGD, X = 0.2, P = 10%) number of species (Table 3, Fig. 4). PTP (maximum likelihood), GMYC and ABGD generally produced similar results with the maximum of 49 species. When the barcoding gap width proxy prior was set to a lower value (X < 1.1), ABGD generally lost sensitivity, inferring 47 or 42 species. Our taxa of interest, the host-specific lineages of *Caparinia*, were inferred as separate species by all *cox*1-based analyses. Similarly, the well-behaved analyses, PTP (maximum likelihood), GMYC, and ABGD, consistently split the American house dust mite, *Dermatophagoides farinae*, into two species, corresponding to *cox*1 groups 1 and 2 (Fig. 4). However, when the X prior was set too low, the prior threshold was high (P ≥ 5.99%) and ABGD lumped *D. farinae* and *D. microceras*. These taxa are similar but reproductively incompatible species, with clear differences in the female spermatheca [40]. Thus, unfortunately, the *cox*1 analyses were not able to infer *D. farinae* within boundaries established by morphological systematics and breeding experiments (Table 3, Fig. 4).

**Table 3:** Summary of 12 species delimitation analyses

Analysis		Loci	Number of species		
			81–taxon alignment	*Caparinia tripilis*+*ictonyctis* (K2P *cox*1 = 7.77%)	*Dermatophagoides farinae* (K2P *cox*1 = 4.19%)
1	PTP Maximum Likelihood	*cox*1	49	2	2
2	bPTP Highest Bayesian supported solution	*cox*1	54	2	6
3	GMYC (3 analyses with different trees)	*cox*1	49	2	2
4	ABGD (X = 1.1; P = 1.29%)	*cox*1	49	2	2
5	ABGD (X = 1.0*–*0.7, 0.5; P = 3.59%)	*cox*1	47	2	2
6	ABGD (X = 0.6, 0.4; P = 5.99%)	*cox*1	42	2	1+
7	ABGD (X = 0.2; P = 10%)	*cox*1	26	1+	1+
8	BPP [θ~G(1,10) τ0~G(1,10)]	5/2 loci	–	1	1
9	BPP [θ~G(2,1000) τ0~G(2,1000)]	5/2 loci	–	1–2	2
10	BPP [θ~G(1,10) τ0~G(2,1000)]	5/2 loci	–	2	2
11	STACEY	5/2 loci	–	2	1
12	PHRAPL	5/2 loci	–	na	2–3

*Key*: 5/2 loci, for *Caparinia*/*Dermatophagoides* datasets, respectively; 5 loci, *18S*+*28S*, *EF1-α*, *SRP54*, *HSP70*, *cox*1 (18S and *28S* were merged because they are linked); 2 loci, *cox*1 and CPW2; 1+, was merged with a closest taxon

*Abbreviation*: na, not available

### Multispecies coalescent species delimitation

Multilocus delimitation analyses based on multispecies coalescent are computationally intensive and, therefore, were run only for our taxa of interest. For the *Caparinia* dataset, BPP analyses suggested lumping *Caparinia ictonyctis* and *C. tripilis* (*s.s.)* into a single species when both ancestral population size and root age are large [θ~G(1,10) τ0~G(1,10)] (Tables 2, 3). This, however, is an unrealistic scenario given a very low prevalence of *Caparinia* in natural host populations (see Additional file 1: Text S1). Under the likely set of priors, small population size and young root age [θ~G(2,1000) τ0~G(2,1000)], the single-species model was only marginally better than *Caparinia* being split into two host-specific species (PP = 0.5142 *vs* 0.4805) (Table 2). Thus, species delimitation is ambiguous here. No single solution, i.e. either one or two species, can be preferred. STACEY, another multispecies coalescent program, agrees with the two-species delimitation scheme of BPP (Table 3). BPP analyses recovered *Dermatophagoides farinae* as one or two species. Under realistic priors, large ancestral effective population size and old root [θ~G(1,10) τ0~G(1,10)], a single-species scenario was preferred (Table 2). STACEY agreed with this delimitation. Surprisingly, PHRAPL did not recover this scenario within a set of top-ranking delimitation models (ΔAIC range 0-2), with the best-fitting single-species model having a ΔAIC of 6.83 (Additional file 9: Table S1). This program extensively relies on “testing” species delimitation models that were initially suggested by the data, thus falling in danger of finding effects that are spurious because random noise is being modeled as structure [41, 42]. In addition, PHRAPL requires estimation of gene trees prior to analysis; so uncertainties in gene tree estimation are not appropriately accounted for, affecting the statistical performance of this method [43].

### Species delimitation in the ‘gray zone’: *Caparinia* and *Dermatophagoides farinae*

The gray zone, an area where conflicting species delimitations are possible, is inherent from the generally continuous nature of the speciation process [44]. However, the typical task of conventional taxonomy is to assign any unknown organism to a species. Considering evidence from analyses based on population genetic theory, STACEY and BPP with realistic priors (small ancestral population and root ages), the two lineages of *Caparinia* may be considered as two separate, host-specific species, *C. tripilis* and *C. ictonyctis*. Similarly, the 7.4–7.8% of *cox*1 sequence divergence (K2P distances), which is well above commonly proposed barcoding thresholds, formally allows these lineages to be considered as separate species (Fig. 4). However, the 7.4–7.8% *cox*1 divergence in the two *Caparinia* lineages is below our ‘conservative’ threshold (> 9.5% or 10.1%, see above). Here we note that these thresholds are based on species having large effective population sizes (*Dermatophagoides farinae* and *Demodex folliculorum*), which makes maintaining high genetic diversity in a population more likely [45], but see [46–48]. In contrast, the host specific lineages of *Caparinia* are expected to have very small population sizes, hence, in these populations, speciation may occur much faster than in large populations due to a larger impact of genetic drift [49, 50]. Furthermore, there are subtle discontinuities in morphological space between the two *Caparinia* lineages (Fig. 1), and their known native ranges do not overlap, indirectly suggesting that these two populations are indeed genetically isolated, although some gene flow between them still cannot be ruled out. Evidence against the two-species scenario is the presence of a very low synonymous + nonsynonymous divergence in nuclear genes: 0.06, 0.09, 0.30 and 0.53% for *28S*, *EF1-α*, *SRP54*, and *HSP70*, respectively (Additional file 5: Table S2). Except for the latter value, this is substantially below the recently proposed genomic ‘gray zone’ based on genomic synonymous divergence, 0.5–2% [51]. Given the above argument we consider the two host-specific lineages as separate species with the caveat that gene flow is possible here. A name for the *Caparinia* species from the African hosts is already available, *Caparinia ictonyctis* Lawrence, 1955 stat. res. Previously, this species was considered as a junior synonym of *Caparinia tripilis* (Michael, 1889) [31].

The American house dust mite, *Dermatophagoides farinae*, is a system that contrasts with the *Caparinia* system in having large population sizes. This species is globally distributed and is common in birds’ nests, suggesting that it had evolved with birds for a relatively long time, whereas its association with humans is a relatively recent event. Yet, this species has a strong *cox*1 genetic structure, forming two distinct *cox*1 lineages, group 1 and 2, with a maximum divergence of 4.2% (Additional file 6: Figure S2) or a minimum distance of 9.3% *versus* its sibling species, *D. microceras* (Additional file 5: Table S2). These species are reproductively isolated and have distinct differences in the female spermatheca [40]. The strong *cox*1 structure observed in *D. farinae* is probably due to past isolation followed by a recent secondary contact; other possible sources of mito-nuclear discordance have been recently reviewed [52]. *Cox*1-only delimitation approaches all suggested that the traditional scope of *D. farinae* is wrong, and it should be split into two or more species, or even be lumped with *D. microceras* when the prior threshold is larger (Fig. 4). Multispecies coalescent-based methods, BPP (assuming large ancestral population size) and STACEY, recovered *D. farinae* as a single species, in agreement with the traditional taxonomy of this species. This is an example of a clear contrast between results of the two approaches and highlights the importance of using demographics in species delimitation.

## Conclusions

Using DNA-based species delimitation analyses has become a common practice in molecular systematics. Most importantly, the *cox*1-barcoding approach has become a standard practice of exploring species boundaries in large datasets. We evaluated several standard species delimitation methods and found that they can produce contradictory results, i.e. the ‘gray’ species delimitation zone, depending on effective population sizes. Populations with large effective sizes can maintain a greater genetic diversity due to their size, which confuses many species delimitation algorithms, resulting in excessive species splitting. This was the case for all species delimitation algorithms, except for STACEY and BPP (only when the population size prior was set appropriately). Particularly, none of the *cox*1-only barcoding analyses were able to delimit correctly our model species with a large effective population size, the American house dust mite, *Dermatophagoides farinae*. In contrast, speciation events are more likely in populations with small effective sizes due to genetic drift/random effects. Overall, many species delimitation algorithms, including *cox*1-only barcoding methods, converge on a single solution here (e.g. two species in the *Caparinia* dataset). Our study, therefore, highlights the importance of using multilocus datasets and incorporating the knowledge of demographic parameters for DNA-based species delimitation analyses.

## Methods

### Material examined

We nearly exhaustively studied available museum collections and collected new specimens. Type and non-type specimen collection information and host data are given in Additional file 1: Text S1. Live mites (*Caparinia* from *Erinaceus europaeus*, ZISP AVB 17-0305-001 and *Atelerix albiventris*, ZISP AVB 14-0505-004, see Additional file 1: Text S1 for more detail) were removed individually using fine and sharp forceps, preserved in 96% ethanol for scanning electron microscopy and molecular analysis or mounted in Hoyer’s medium [53]. House dust mite datasets (Additional file 2: Table S1) were described previously [54, 55]. For the purpose of this work we consider that census population size and effective population size are highly correlated. Everything else being equal, a species with a small census population size will also have a small effective population size, while a species with a large census population size will likely have a large effective population size (e.g. *Dermatophagoides farinae*) relative to the rare species (e.g. *Caparinia*).

### DNA amplification, sequencing and alignment

We sequenced individual specimens of *Caparinia* from *Atelerix albiventris* and *Erinaceus europaeus* for 6 genes: two nuclear ribosomal RNA genes, *18S* and *28S* rDNA; three nuclear protein-coding genes: elongation factor 1alpha100E (*EF1-α*), signal recognition particle protein 54k (*SRP54*), *Hsc70-5* heat shock protein cognate 5 (here abbreviated as *HSP70*); and one mitochondrial protein-coding gene (*cox*1). *Cox*1 was sequenced from 14 specimens for *Caparinia* ex *Atelerix albiventris* (all were identical) and 2 specimens of *Caparinia tripilis* ex *Erinaceus europaeus*. We used previously published amplification and sequencing protocols [56–59]. To serve as a reference, populations of *Dermatophagoides farinae* and *Dermatophagoides pteronyssinus* from both Old and New World populations were sequenced for *cox*1 and the nuclear cysteine proteinase-1 preproenzyme gene (*CPW2*, encoding the major group 1 house dust allergen, abbreviated as *Der f1* and *Der p1* for the two species, respectively). Primers, amplification, and sequencing of this gene were described previously [54]. GenBank accession numbers are as follows: MG766225-MG766259, MG766261-MG766269 (Additional file 2: Table S1). The sequence of *18S* of *Caparinia* from *Erinaceus europaeus* (GenBank: MG766260) was identified as a gregarine (an endoparasitic protozoan) and, therefore, was excluded from further analyses. Domain D4 of *28S* rDNA was also excluded because our standard protocol produced superimposed sequences. rDNA sequences were aligned in Mesquite ver. 3.31 [60] using a previously established secondary structure model [59]; alignment of other loci was unambiguous. Voucher and co-voucher mite specimens are deposited in the University of Michigan Museum of Zoology, Ann Arbor, Michigan under the following accession numbers: *Caparinia ictonyctis* ex *Atelerix albiventris* [BMOC 13-0508-003 (AD1647)]; *Caparinia tripilis* ex *Erinaceus europaeus* [BMOC 16-0825-012 (AD2034); BMOC 16-0825-013 (AD2035)].

### Evaluation of the quality of GenBank sequences

Sequences deposited in public repositories, such as GenBank, may contain (i) sequencing errors or artifacts (e.g. unnoticed polymerase errors introduced as part of molecular cloning, using low-quality sequence data, or vector/primer sequence contamination); (ii) inaccurate morphology-based identification; (iii) sample contamination or mislabeling. For Pyroglyphidae, we downloaded the available *cox*1 sequences (GenBank databases: nucleotide, whole genome shotgun contigs, expressed sequence tag) and evaluated their quality using our reference sequences from our specimens carefully identified using morphology. We color-coded our alignment by amino acid transition, and then we looked for unusual amino acid substitutions, stop codons, and frameshifting indels. Maximum likelihood trees with and without the problematic sequences were constructed to see if these sequences could affect phylogenetic inference (Additional file 3: Figure S1, Additional file 4: Alignment S1). For Psoroptidae, we included 12 GenBank sequences, six of which were trimmed to exclude unusual substitutions and frameshifting deletions at the 3’ end as described previously [57].

### Genetic distances

Following tradition, we use Kimura 2-parameter (K2P) genetic distances [2], but see [61] for criticism. Distances were calculated in PAUP* ver 4.0a (build 158) [62] using the default settings. Uncorrected p-distances were also calculated for comparison; we did not identify extreme K2P values in comparison to uncorrected p-distances, hence we do not report the latter further. We also calculated uncorrected amino acid distances for the same DNA sequences in PAUP. These distances were multiplied by 3 to be compatible with K2P distances on the same plot. To give a morphological context to genetic distance values, taxa were scored as having “clear” (no overlap), “weak” (can mostly be separated morphologically, but with an overlap), or “no” morphological differences from a sister taxon (Additional file 5: Table S2).

### Phylogenetic inference

Substitution models and best partitioning strategies were estimated in PartitionFinder v1.1.1 [63]. The best partitioning scheme contained 7 loci and 4 partitions: *18S* stem + *28* stem (TVM+I+G), *18S* loop + *28S* loop (TIM+I+G), *EF1-α* + *SRP54* + *HSP70* + *CPW2* (GTR+I+G), *cox*1 (GTR+I+G). An alignment containing 86 individuals classified in 42 morphospecies was used (Additional file 2: Table S1). Phylogenetic relationships were inferred in a Bayesian framework in BEAST 2.4.7, with the clock model set to “Relaxed Clock Log Normal”, Yule speciation model, 7.6*10^7^ generations, and a 17% ‘burn-in’ as determined by examining ESS values and trace graphs in Tracer. Six independent analyses were run to confirm convergence. A similar maximum likelihood tree was also inferred in RAxML v.8.2.9 [64] (not reported further).

### DNA-based species delimitation

We explored several methods of species delimitation, each making different assumptions (see the Introduction section above): threshold-based ABGD [14]; multispecies coalescent-based; BPP v3.3 [21, 65, 66]; STACEY [22]; PHRAPL [23], and other commonly used species delimitation algorithms, GMYC [24] and PTP [25].

#### GMYC

This method uses an ultrametric tree and attempts to detect the transition in the tree where the branching pattern switches from being attributed to speciation (one lineage per species) to when it can be attributed to the intra-species coalescent process (multiple lineages per species) [24]. GMYC infers a single cut-off time T where all nodes above T represent species; a multi-threshold algorithm is available but it is less accurate [67, 68]. To evaluate sensitivity of the method to different assumptions related to tree priors, we inferred several ultrametric topologies in BEAST using different sets of priors on tree branching and the rate of molecular evolution: a Yule model and a constant clock; a Yule model and a relaxed clock; a coalescent model with constant population size and a constant clock. All BEAST analyses used the GTR+I+G model of nucleotide substitution suggested by the program PartitionFinder. Species delimitation analyses were run using the function gmyc of the R package *splits* v.1.0-19 [69]. For these analyses, we used an 81-taxon, 1238 nt *cox*1 alignment.

#### PTP

This method is similar to GMYC, but it uses branch lengths expressed in terms of nucleotide substitutions, rather than in time units, as required by GMYC. We ran PTP using the webserver (https://cme.h-its.org/exelixis/web/software/PTP/), which includes both the original maximum likelihood version and the updated Bayesian version (bPTP). Here we used the same dataset as for GMYC analyses (see above) and a phylogenetic tree inferred in RAxML using the GTR+I+G model of nucleotide substitution.

#### ABGD

This program uses a range of prior intraspecific divergences (P) to infer a model-based, one-sided confidence limit for within-species divergence. Then the method detects the barcode gap as the first significant gap beyond this limit and uses it to partition the data. Inference of the limit and gap detection are then recursively applied to previously obtained groups to get finer partitions until no further partitioning occurs [14]. No prior knowledge on species boundaries is required, which is a great advantage of this program. The command-line version of the program was run as follows: “./abgd -a -d 0 -X 0.6 *.fas”; where -a = output all partitions and tree files; -d 0 = computes a matrix of pairwise K2P distances; -X = proxy for the minimum gap width, using the default, X = 1.5, or any value above 1.1, was impossible because the program did not find more than one partition, so this parameter was varied between 1.1–0.2 by an increment of -0.1 (10 analyses total); and *.fas = input sequence alignment in fasta format. Other parameter values were defaults. P was varied by the program from 0.001 to 0.100, which is the default. Because ABGD requires a large training dataset, we employed an 81-taxon 1238 nt *cox*1 alignment (same as for GMYC analyses), containing our target taxa (*Caparinia*, *Dermatophagoides farinae*), as well as other mites of the two related families, Pyroglyphidae and Psoroptidae, plus outgroups.

#### BPP

To evaluate the influence of the ancestral population size (θ) and root age (τ0) priors on the posterior probabilities of species models, we used three combinations of priors [20]: set1: θ ~ G(1, 10) τ0 ~ G(1, 10); set2: θ ~ G(2, 1000) τ0 ~ G(2, 1000); and set3: θ ~ G(1, 10) τ0 ~ G(2, 1000). Other divergence time parameters were assigned the Dirichlet prior (equation 2 in [66]). Set1 assumes large values for both θ and τ0; Set2 assumes small values for both θ and τ0; while Set3 assumes large values for θ and small values for τ0, favoring conservative models containing fewer species [66]. We used the automatic MCMC fine-tune method for the *Dermatophagoides* dataset, while for the *Caparinia* dataset adjustment of finetune variables was necessary. The adjustment was done so that the acceptance proportions are close to 0.3 or lie in the interval (0.15–0.7). For each of the two datasets and each combination of the priors, we conducted two separate analyses: (i) estimating the θ and τ parameters (A00: speciesdelimitation = 0, speciestree = 0) using a tree inferred in RAxML as the guide tree; and (ii) combined species delimitation and species tree inference (A11: speciesdelimitation = 1, speciestree = 1) with reversible jump (rjMCMC). The heredity scalar was set to 1 (nuclear genes) or 0.25 (mitochondrial genes). All analyses were run for 100,000 generations and a sampling frequency of 1; the first 4000 MCMC samples were discarded as ‘burn-in’. Marginal likelihoods (Bayes factors) were calculated in BFdriver included in the BPP package; the number of points in the Gauss-Legendre quadrature algorithm for numerical integration was set to K = 16. For the *Caparinia* dataset we used 5 presumably unlinked loci (*18S*+*28S*, *EF1-α*, *SRP54*, *HSP70* and *cox*1) and three putative OTUs: *Otodectes cynotis* (Ocy), *Caparinia ictonyctis* from captive *Atelerix albiventris* (Caic) and *Caparinia tripilis* from *Erinaceus europaeus* (Catri). For the *Dermatophagoides* dataset we used 2 loci (*cox*1 and *CPW2*) and four putative OTUs: *Dermatophagoides pteronyssinus* (DP), *D. microceras* (DM), and *D. farinae* group 1 (DFa) and group 2 (DFb).

#### STACEY

This program is based on multispecies coalescent as implemented in *BEAST [70] but uses an extension of this model called the birth-death-collapse model [22]. This model assumes a priori “minimal clusters” of individuals, which can be merged, but not split by the program. There are several priors specific to species delimitation. Most importantly, the Collapse Weight prior provides information about the likely number of species in a delimitation analysis, where values near 1 mean fewer species. In our analyses, the Collapse Weight prior was estimated and set to a uniform distribution [0, 1]. For the *Caparinia* dataset, the following models of nucleotide substitution were set for five presumably unlinked loci: TIM (rDNA); TrN (*EF1-α*); TVM+G (*SRP54*); TrN+G (*HSP70*); and TVM+I (*cox*1). For the two-locus *Dermatophagoides* dataset, models were as follows: HKY+G (*cox*1); TIM+G (*CPW2*). STACEY was run with the strict clock model; the coalescent parameters were set as suggested in the STACEY manual v1.2.3; MCMC chain length was set to 10^9^ sampling every 10^6^ generation; 4–7 independent analyses were run to ensure consistency between runs. Runs that converged on a similar distribution were combined. Convergence, mixing, and ESSs were estimated in Tracer v1.6 [71]. For the *Caparinia* dataset, we evaluated single- and two-species models where *Caparinia ictonyctis* was either merged with *Caparinia tripilis* s. str. to form a ‘minimal cluster’ or these two OTUs were treated separately (see the BPP section above). For the *Dermatophagoides* dataset, we tested whether *Dermatophagoides farinae* groups 1 and 2 (DFa, DFb) are one or two species (see the BPP section above). In addition, because of the presence of a large number of individuals, we ran a species discovery analysis, where each individual was treated as a separate ‘minimal cluster’. Model comparison was done by using marginal likelihoods (Bayes factors); with standard errors estimated from 16–100 bootstrap replicates in Tracer [71].

#### PHRAPL

Because PHRAPL needs at least 3 *a priori* groups to run, and because the sequence of *CTW2* for *D. microceras* (an outgroup) was not available, we split *Dermatophagoides farinae* group 2 into two shallow subgroups, B and C (Additional file 6: Figure S2). Of these, group C was a monophyletic, *cox*1-only lineage (not recovered by *CPW2*). Gene trees were inferred in RAxML, and then they were rooted to mid-point in the R package *phangron* [72]. PHRAPL was run with a tip subsampling of 3 tips per 3 populations [popAssignments<-list(c(3,3,3)))], no outgroup (outgroup=FALSE, outgroupPrune=FALSE), and modelRange=1:48 (i.e. all 48 models available in migrationArray); other settings were left at default. We calculated genealogical divergence index (gdi), a composite metric that estimates overall divergence (between 0 and 1) from the combined effects of genetic drift and gene flow, where gdi = 0 corresponds to panmictic populatuons, while gdi = 1 corresponds to strong divergence (speciation). It was not possible to run a PHRAPL analysis for *Caparinia* because there were putative OTUs with fewer than 2 individuals.

For model-based analyses, equivalence of models was established as the following rough rule of thumb [73]: substantial (ΔAIC = 0–2); weak (ΔAIC = 4–7); none (ΔAIC >10). For model comparison using marginal likelihoods (Bayesian factors, BF), the following scale was used [74]: BF = 0–2 (not worth more than a bare mention); BF = 2–6 (positive evidence); BF = 6–10 (strong support); and BF > 10 (decisive).

## Additional files

Additional file 1: **Text S1.** Material studied. (DOCX 19 kb)

Additional file 2: **Table S1.** Taxa, collection data, and GenBank accession numbers. (DOCX 44 kb)

Additional file 3: **Figure S1.** Position of low quality sequences of two species of *Dermatophagoides* on phylogenetic tree. Low quality sequences are identified in nexus file S4 (amino acid color-coded alignment should be viewed in the program Mesquite). (PDF 164 kb)

Additional file 4: **Alignment S1.** Alignment of sequences generated in this study and GenBank sequences of *Dermatophagoides*. Low quality sequences are identified by amino-acid color-coding (should be viewed in the program Mesquite). (NEX 80 kb)

Additional file 5: **Table S2.** Within- and among-species genetic distances of 7 loci of 10 putative species. (DOCX 79 kb)

Additional file 6: **Figure S2.** *Dermatophagoides farinae* CO1 and CPW2 gene trees inferred in a Maximum Likelihood framework (RAxML). Important groupings are indicated. (PDF 215 kb)

Additional file 7: **Table S3.** Summary of species delimitation analyses. (XLSX 22 kb)

Additional file 8: **Figure S3.** Similarity matrix of STACEY species discovery analysis of the *Dermatophagoides* dataset. (PDF 138 kb)

Additional file 9: **Table S4.** Summary of PHRAPL analyses of the *Dermatophagoides* dataset. (XLSX 90 kb)

Additional file 10: **Alignment S2.** Nexus datamatrix of aligned sequences of seven loci used to infer species tree (Fig. 4). Original contiguous alignments as well as final partitions (identified by the suffix _noee») used in analyses are given. The file should be viewed in the program Mesquite. (NEX 7275 kb)

## Abbreviations

G: Gamma parameter
I: Invariant site parameter
*18S*: Small subunit ribosomal RNA gene
*28S*: Large subunit ribosomal RNA gene
rDNA: Ribosomal DNA
ABGD: Automatic Barcode Gap Discovery (species delimitation algorithm)
AIC: Akaike information criterion
BEAST: Bayesian Evolutionary Analysis Sampling Trees (phylogenetic tree inference program)
BF: Bayes Factor
BPP: Bayesian Phylogenetics and Phylogeography (species delimitation algorithm)
bPTP: Bayesian Poisson Tree Processes model (species delimitation algorithm)
*cox*1: Cytochrome *c* oxidase subunit 1 gene
*CPW2*: cysteine protease precursor (this locus has specific abbreviations for each species
Der p1: Group 1 allergen preproenzyme for *Dermatophagoides pteronyssinus*
Der f 1: Group 1 allergen preproenzyme for *D. farinae*
*EF1-α*: Elongation factor 1alpha100E gene
ESS: Effective sample size of MCMC (Markov chain Monte Carlo)
gdi: Genealogical divergence index
GMYC: Generalized Mixed Yule Coalescent (species delimitation algorithm)
GTR: General time reversible model of nucleotide evolution
HKY: Hasegawa, Kishino & Yano, 1985 model of nucleotide evolution
*HSP70*: heat-shock protein cognate 5 gene
K2P: Kimura 2-parameter)
OTU: Operational taxonomic unit
PAUP*: Phylogenetic Analysis Using Parsimony and Other Methods (phylogenetic tree inference program)
PHRAPL: Phylogeography using Approximate Likelihood (species delimitation algorithm)
PTP: Poisson Tree Processes model (species delimitation algorithm)
RAxML: Randomized Accelerated Maximum Likelihood (phylogenetic tree inference program)
rjMCMC: reversible jump Markov chain Monte Carlo
*SRP54*: Signal recognition particle protein 54k gene
STACEY: Species Tree And Classification Estimation, Yarely (species delimitation and phylogeny estimation algorithm)
TIM: transitional model of nucleotide evolution
TrN: Tamura & Nei model of nucleotide evolution
TVM: transversional model of nucleotide evolution
X: barcode gap width proxy (applies to ABGD)
Δ: change of a value
θ: ancestral population size
τ: root age
