# Supplementary material for: Cox1 barcoding versus multilocus species delimitation: validation of two mite species with contrasting effective population sizes
Source: Parasit Vectors. 2019 Jan 5;12:8. doi: 10.1186/s13071-018-3242-5 (PMC6321676; doi:10.1186/s13071-018-3242-5)
Supplement: Supplementary file 1 — Text S1. Material studied. (DOCX 19 kb) [file 13071_2018_3242_MOESM1_ESM.docx]

**Additional file 1. Material studied**

***Caparinia tripilis* (Michael, 1889)**

ex *Erinaceus europaeus*: 1 male and 1 tritonymph, **Spain**, Madrid Province, San Fernando de Henares, 40°25'25''N, 3°31'57''W, 28 June 1977, coll. M. Portus (IRSNB); 1 male, 3 females, 4 tritonymphs, 2 larvae, the **Netherlands**, Nijmegen, 51°50'33''N, 5°51'10''E, 29 August 1967, coll. F.S. Lukoschus (IRSNB); 6 males, 2 females, and 2 tritonymphs, Belgium, Flemish Brabant Province, Leefdaal, 50°50'51''N, 4°35'21''E, 11 July 1979, coll. F. Puylaert (IRSNB); 1 female, **Poland**, Kraków, 50°5'N, 19°55'E, 17 July 1966, coll. Zurowski (IRSNB); 20 males, 20 females, 10 tritonymphs, 10 protonymphs, and 10 larvae, **Russia**, St.-Petersburg Province, Gatchina District, near Taytsy village, 59°40'00''N, 40°07'00''E, September 2015, coll. A.V. Bochkov (ZISP, AVB 17-0305-001) [many additional specimens are preserved in alcohol or were used for scanning electron microscopy and molecular analysis]; 10 males, 10 females, 10 tritonymphs, 10 protonymphs, and 10 larvae, Russia, St. Petersburg Province, no further data, 05 March 1988, coll. I. Skiba (ZISP AVB 14-0505-008).

The holotype specimen of *Caparinia tripilis,* BMNH 1930.8.25.2828 labeled “*Symbiotes tripilis*, hedgehog, 88, A.D. Michael” housed in the Natural History Museum, London, England was not examined.

ex *Erinaceus* *roumanicus*: 10 males, 10 females, 10 tritonymphs, 10 protonymphs, and 10 larvae, **Russia**, North Caucasus, Caucasus national Reserve, Khosta forest, 23 June 1977, coll. H. Dubinina (ZISP). Note. The original label says *Erinaceus europaeus*, however, based on the locality, the host name should be *E.* *roumanicus* (He et al. 2012).

***Caparinia ictonyctis* (Lawrence, 1955)**

ex *Atelerix albiventris*: 20 males, 20 females, 10 tritonymphs, 10 protonymphs, and 10 larvae, **South Korea**, South Leolla Province, Gwangju, pet shop, February 2010, coll. S.-S. Shin (ZISP AVB 14-0505-004) [many additional specimens are preserved in alcohol or were used for scanning electron microscopy and molecular analysis]; 6 males and 1 female, **USA**, Maryland, Baltimore Zoo, directly transported from Togo, 4 February 1986, coll. E. Neely (IRSNB).



ex *Ictonyx striatus*: 3 males and 3 females (syntypes of *Caparinia ictonyctis* Lawrence, 1955), **South Africa**, KwaZulu-Natal, Pietermaritzburg, 29°27'00''S, 30°23'00''E, April, 1954, coll. R.F. Lawrence (KZNM 5767); 1 male and 1 female, [original label on French: *Ictonyx striatus* *erythreae*, Egypte, Saukin], other data unknown (IRSNB). On the original label by A. Fain, the geographical data are probably not quite correct. *Ictonyx striatus* *erythreae* is absent in Egypt but occurs in neighbouring **Sudan** [1]. The locality “Saukin” is absent in Egypt and according to Geonames database probably is El Qadarif city, 14°02'05''N, 35°23'00''E, Al Qadarif state, Sennar Province, Sudan.

**Abbreviations**

**BMNH**: the Natural History Museum, London, England **IRSNB**: Institut Royal des Sciences Naturelles de Belgique, Brussels, Belgium **KZNM**: KwaZulu-Natal Muzeum, Pietermaritzburg, South Africa **ZISP**: Zoological Institute of the Russian Academy of Sciences, Saint Petersburg, Russia.

**References**

1. Wilson DE, Reeder DM: **Mammal Species of the World. A Taxonomic and Geographic Reference (3rd ed)**. Baltimore: Johns Hopkins University Press; 2005.
