# Supplementary material for: Cox1 barcoding versus multilocus species delimitation: validation of two mite species with contrasting effective population sizes
Source: Parasit Vectors. 2019 Jan 5;12:8. doi: 10.1186/s13071-018-3242-5 (PMC6321676; doi:10.1186/s13071-018-3242-5)
Supplement: Supplementary file 2 — Table S1. Taxa, collection data, and GenBank accession numbers. (DOCX 44 kb) [file 13071_2018_3242_MOESM2_ESM.docx]

**Additional file 2: Table S1**. Taxa, collection data, and GenBank accession numbers (id=unique numbers for DNA extraction vouchers; field number = lot-based museum accession numbers for vouchers and co-vouchers).

| **id** | **Mite species** | **Mite family** | **Host** | **Country** | **filed number** | **18S** | **28S** | **EF1-α** | **SRP54** | **HSP70** | **CO1** | **CPW2** |
| --- | --- | --- | --- | --- | --- | --- | --- | --- | --- | --- | --- | --- |
| 1028 | Hyomesalges sp. | Psoroptoididae | Tockus pallidirostris | MALAWI | BMOC 07-1229-001 | JQ000220 | JQ000528 | JQ000830 | JQ001133 | JQ001448 | KF891925 | - |
| 1042 | Mesalgoides sp. | Psoroptoididae | Spizella passerina | USA | BMOC 06-0717-001 | JQ000233 | JQ000541 | JQ000842 | JQ001146 | JQ001461 | MG766229 | - |
| 1111 | Hirstia chelidonis | Pyroglyphidae | - | FRANCE | BMOC 08-0103-001 | JQ000245 | JQ000553 | JQ000854 | JQ001158 | JQ001473 | KF891939 | - |
| 1127 | Myialges sp. | Epidermoptidae | Hippoboscidae | KAZAKHSTAN | BMOC 07-1119-039 | JQ000255 | JQ000563 | JQ000863 | JQ001168 | JQ001483 | MG766230 | - |
| 1135 | Mouchetia aff indochinensis | Pteronyssidae | Zosterops erythropleurus | RUSSIA | BMOC 08-0608-008 | JQ000175 | JQ000483 | JQ000786 | JQ001088 | JQ001403 | KF891919 | - |
| 1152 | Picalgoides_aff_pteroglossorum | Psoroptoididae | Ramphastos sulfuratus | MEXICO | BMOC 08-0515-083 | JQ000224 | JQ000532 | JQ000834 | JQ001137 | JQ001452 | KF891928 | - |
| 1162 | gen. aff. Paralgopsis | Pyroglyphidae | Melanerpes aurifrons | MEXICO | BMOC 08-0515-085 | JQ000235 | JQ000543 | JQ000844 | JQ001148 | JQ001463 | MG766231 | - |
| 1163 | Paralgopsis sp. | Pyroglyphidae | Amazona autumnalis | MEXICO | BMOC 08-0515-092 | JQ000236 | JQ000544 | JQ000845 | JQ001149 | JQ001464 | KP406746 | - |
| 1171 | Ptyssalges sp. | Ptyssalgidae | Campylopterus excellens | MEXICO | BMOC 08-0515-098 | JQ000189 | JQ000497 | JQ000800 | JQ001102 | JQ001417 | KY922489 | - |
| 1177 | Promyialges sp. | Epidermoptidae | Euphonia hirundinacea | MEXICO | BMOC 08-0515-073 | JQ000256 | JQ000564 | JQ000864 | JQ001169 | JQ001484 | KY922492 | - |
| 1190 | Microlichus sp. | Epidermoptidae | Amazilia tzacatl | MEXICO | BMOC 08-0515-101 | JQ000254 | JQ000562 | JQ000862 | JQ001167 | JQ001482 | KY922491 | - |
| 1197 | Pandalura sp. | Psoroptoididae | Ciccaba virgata | MEXICO | BMOC 08-0515-117 | KF891889 | KF891897 | KF891903 | KF891909 | KF891915 | KF891923 | - |
| 1210 | Dermatophagoides cf. alexfaini | Pyroglyphidae | Hirundo rustica erythrogaster | MEXICO | BMOC 08-0515-160 | JQ000248 | JQ000556 | JQ000857 | JQ001161 | JQ001476 | KF891941 | - |
| 1412 | Euroglyphus maynei | Pyroglyphidae | - | USA | BMOC 08-1120-001 | JQ000244 | JQ000552 | JQ000853 | JQ001157 | JQ001472 | KP406747 | - |
| 1416 | Turbinoptes strandtmanni | Turbinoptidae | Larus delawarensis | USA | BMOC 09-1202-001 | MG766225 | MG766267 | MG766263 | MG766265 | MG766228 | - | - |
| 1418 | Onychalges sp2 | Pyroglyphidae | Lagonosticta rhodopareia | MALAWI | BMOC 10-0503-002 | JQ000251 | JQ000559 | JQ000860 | JQ001164 | JQ001479 | KP406750 | - |
| 1419 | Onychalges pachyspathus | Pyroglyphidae | Estrilda astrild | MALAWI | BMOC 10-0503-003 | JQ000252 | JQ000560 | JQ000861 | JQ001165 | JQ001480 | KP406751 | - |
| 1427 | Dermatophagoides pteronyssinus | Pyroglyphidae |  | SINGAPORE | BMOC 08-0912-029 | JQ000249 | JQ000557 | JQ000858 | JQ001162 | JQ001477 | KP406749 | - |
| 1458 | Psoralges libertus | Psoroptidae | Tamandua sp. | USA | BMOC 12-0128-001 | KF891890 | KF891898 | - | - | - | KF891932 | - |
| 1647 | Caparinia ictonyctis | Psoroptidae | Atelerix albiventris | SOUTH KOREA | BMOC 13-0508-003 | KF891891 | MG766268 | MG766261 | MG766266 | MG766227 | KF891934 | - |
| 1660 | Chorioptes bovis | Psoroptidae | Ovis aries | ICELAND | BMOC 13-0810-007 | KF891892 | KF891899 | KF891904 | KF891910 | KF891916 | KF891935 | - |
| 1664 | Chorioptes sweatmani | Psoroptidae | Alces alces | SWEDEN | BMOC 13-0810-010 | KF891893 | KP406736 | KF891905 | KF891911 | KF891917 | KF891936 | - |
| 1753 | Dermatophagoides microceras | Pyroglyphidae | - | SPAIN | BMOC 14-0614-019 | KP406743 | KP406737 | KP406734 | KP406741 | KP406739 | MG766232 | - |
| 2034 | Caparinia tripilis | Psoroptidae | Erinaceus europaeus | RUSSIA | BMOC 16-0825-012 | -* | MG766269 | MG766262 | MG766264 | MG766226 | MG766233 | - |
| 2035 | Caparinia tripilis | Psoroptidae | Erinaceus europaeus | RUSSIA | BMOC 16-0825-013 | - | - | - | - | - | MG766234 | - |
| 466 | Otodectes cynotis | Psoroptidae | Felis catus | USA | BMOC 04-1208-001 | JQ000240 | JQ000548 | JQ000849 | JQ001153 | JQ001468 | KF891933 | - |
| 515 | Sturnophagoides bakeri | Pyroglyphidae | Progne subis nest | USA | BMOC 05-0814-001 | JQ000246 | JQ000554 | JQ000855 | JQ001159 | JQ001474 | KF891940 | - |
| 521 | Dermatophagoides farinae | Pyroglyphidae | Dermestes maculatus culture | USA | BMOC 05-0812-001 | JQ000247 | JQ000555 | JQ000856 | JQ001160 | JQ001475 | - | - |
| 538 | Schoutedenocoptes aquilae | Turbinoptidae | Geranospiza caerulescens | PANAMA | BMOC 05-1006-001 | JQ000237 | JQ000545 | JQ000846 | JQ001150 | JQ001465 | - | - |
| 572 | Echimytricalges guyanensis | Lobalgidae | Proechimys simonsi, Peru | PERU | BMOC 02-0919-017 | JQ000239 | JQ000547 | JQ000848 | JQ001152 | JQ001467 | KF891931 | - |
| 582 | Picalgoides sp. n. | Psoroptoididae | Colaptes auratus | USA | BMOC 06-0504-001 | JQ000223 | JQ000531 | JQ000833 | JQ001136 | JQ001451 | KF891927 | - |
| 597 | Psoroptes ovis | Psoroptidae | Oryctolagus cuniculus | USA | BMOC 05-0105-001 | JQ000241 | JQ000549 | JQ000850 | JQ001154 | JQ001469 | - | - |
| 597 | Psoroptes ovis | Psoroptidae | Oryctolagus cuniculus | USA | BMOC 05-0105-001 | JQ000241 | JQ000549 | JQ000850 | JQ001154 | JQ001469 | KJ957822 | - |
| 637 | Mesalgoides sp. | Psoroptoididae | Sheppardia bocagei | TANZANIA | BMOC 03-0831-023 | JQ000229 | JQ000537 | JQ000838 | JQ001142 | JQ001457 | KF891930 | - |
| 675 | Scutulanyssus subis | Pteronyssidae | Progne subis | USA | BMOC 06-0706-001 | JQ000185 | JQ000493 | JQ000796 | JQ001098 | JQ001413 | KF891920 | - |
| 676 | Scutulanyssus obscurus | Pteronyssidae | Hirundo rustica | USA | BMOC 06-0612-026 | JQ000186 | JQ000494 | JQ000797 | JQ001099 | JQ001414 | KU203089 | - |
| 707 | Laronyssus marinus | Avenzoariidae | Larus cachinnans mongolicus | RUSSIA | BMOC 06-0910-068 | KF891886 | KF891894 | KF891900 | KF891906 | KF891912 | KF891918 | - |
| 716 | Pandalura oconnori | Psoroptoididae | Steatornis caripensis | PERU | BMOC 06-1124-001 | KF891887 | KF891895 | KF891901 | KF891907 | KF891913 | KF891921 | - |
| 733 | gen. aff. Paralgopsis | Pyroglyphidae | Colaptes auratus | USA | BMOC 06-1117-016 | JQ000234 | JQ000542 | JQ000843 | JQ001147 | JQ001462 | KP406745 | - |
| 749 | Pandalura strigisoti | Psoroptoididae | Asio otus | RUSSIA | BMOC 06-1119-114 | KF891888 | KF891896 | KF891902 | KF891908 | KF891914 | KF891922 | - |
| 757 | Picalgoides picimajoris | Psoroptoididae | Dendrocopos major, Russia | RUSSIA | BMOC 06-1119-177 | JQ000222 | JQ000530 | JQ000832 | JQ001135 | JQ001450 | KF891926 | - |
| 759 | Microlichus sp | Epidermoptidae | Hirundo rustica | RUSSIA | BMOC 06-1119-228 | JQ000253 | JQ000561 | EU152823 | JQ001166 | JQ001481 | MG766235 | - |
| 773 | Mesalgoides sp. n. | Psoroptoididae | Grallaria capitalis | PERU | BMOC 06-0924-024 | JQ000228 | JQ000536 | EU152818 | JQ001141 | JQ001456 | KF891929 | - |
| 796 | Congocoptes sphyrapicicola | Turbinoptidae | Sphyrapicus varius, IL | USA | BMOC 07-0220-010 | JQ000238 | JQ000546 | JQ000847 | JQ001151 | JQ001466 | KY922490 | - |
| 843 | Gymnoglyphus longior | Pyroglyphidae | - | RUSSIA | BMOC 07-0815-031 | JQ000242 | JQ000550 | JQ000851 | JQ001155 | JQ001470 | KF891937 | - |
| 903 | Temnalges sp. | Psoroptoididae | Gallinula chloropus, MI | USA | BMOC 07-0530-005 | JQ000219 | JQ000527 | JQ000829 | JQ001132 | JQ001447 | KF891924 | - |
| 947 | Dermatophagoides evansi | Pyroglyphidae | Hirundo rustica | USA | BMOC 07-0607-007 | JQ000250 | JQ000558 | JQ000859 | JQ001163 | JQ001478 | KF891942 | - |
| 949 | Gymnoglyphus osu | Pyroglyphidae | - | USA | BMOC 07-0607-008 | JQ000243 | JQ000551 | JQ000852 | JQ001156 | JQ001471 | KF891938 | - |
| - | Chorioptes bovis | Psoroptoididae | Bison bonasus | POLAND | - | GQ864308 | - | - | - | - | - | - |
| - | Chorioptes panda | Psoroptoididae | Ailuropoda melanoleuca | CHINA | - | FJ907511 | - | - | - | - | - | - |
| - | Chorioptes texanus | Psoroptoididae | Bos taurus | CHINA | - | FJ907507 | - | - | - | - | - | - |
| - | Chorioptes texanus | Psoroptoididae | Bos taurus | CHINA | - | FJ907508 | - | - | - | - | - | - |
| - | Chorioptes texanus | Psoroptoididae | Bos taurus | CHINA | - | FJ907509 | - | - | - | - | - | - |
| - | Chorioptes texanus | Psoroptoididae | Bos taurus | CHINA | - | FJ907510 | - | - | - | - | - | - |
| - | Dermatophagoides pteronyssinus | Pyroglyphidae | - | BELGIUM | - | - | - | - | - | - | EU884425 | - |
| - | Euroglyphus maynei | Pyroglyphidae | - | USA | - | - | - | - | - | - | MUJZ01072749 | - |
| - | Otodectes cynotis | Psoroptoididae | Felis catus | POLAND | - | GQ864320 | - | - | - | - | - | - |
| - | Psoroptes natalensis | Psoroptoididae | Bubalus bubalis | CHINA | - | - | - | - | - | - | GQ221770 | - |
| - | Psoroptes ovis | Psoroptoididae | Oryctolagus cuniculus | CHINA | - | - | - | - | - | - | KJ957822 | - |
| - | Psoroptes ovis | #N/A | Oryctolagus cuniculus | CHINA | - | FJ907505 | - | - | - | - | FJ907499 | - |
| - | Psoroptes ovis | #N/A | Ovis aries | UNITED KINGDOM | - | - | - | - | - | - | BQ835080 | - |
| - | Psoroptes ovis | #N/A | Ovis aries | UNITED KINGDOM | - | - | - | - | - | - | FR748605 | - |
| 521.RS03 | Dermatophagoides farinae | Pyroglyphidae | - | USA | BMOC 05-0812-001 | - | - | - | - | - | MG766236 | KJ542064 |
| 521.RS59 | Dermatophagoides farinae | Pyroglyphidae | - | USA | BMOC 05-0812-001 | - | - | - | - | - | MG766237 | KJ542081 |
| 521.RS60 | Dermatophagoides farinae | Pyroglyphidae | - | USA | BMOC 05-0812-001 | - | - | - | - | - | MG766238 | KJ542082 |
| 521.RS61 | Dermatophagoides farinae | Pyroglyphidae | - | USA | BMOC 05-0812-001 | - | - | - | - | - | MG766239 | KJ542083 |
| 521.RS62 | Dermatophagoides farinae | Pyroglyphidae | - | USA | BMOC 05-0812-001 | - | - | - | - | - | MG766240 | KJ542084 |
| 521.RS63 | Dermatophagoides farinae | Pyroglyphidae | - | USA | BMOC 05-0812-001 | - | - | - | - | - | MG766241 | KJ542085 |
| 1709.RS17 | Dermatophagoides farinae | Pyroglyphidae | - | PAKISTAN | BMOC 12-0723-002 | - | - | - | - | - | MG766242 | KJ542065 |
| 1709.RS20 | Dermatophagoides farinae | Pyroglyphidae | - | PAKISTAN | BMOC 12-0723-002 | - | - | - | - | - | MG766243 | KJ542066 |
| 1709.RS26 | Dermatophagoides farinae | Pyroglyphidae | - | PAKISTAN | BMOC 12-0723-002 | - | - | - | - | - | MG766244 | KJ542068 |
| 1709.RS27 | Dermatophagoides farinae | Pyroglyphidae | - | PAKISTAN | BMOC 12-0723-002 | - | - | - | - | - | MG766245 | KJ542069 |
| 1709.RS30 | Dermatophagoides farinae | Pyroglyphidae | - | PAKISTAN | BMOC 12-0723-002 | - | - | - | - | - | MG766246 | KJ542071 |
| 1709.RS33 | Dermatophagoides farinae | Pyroglyphidae | - | PAKISTAN | BMOC 12-0723-002 | - | - | - | - | - | MG766247 | KJ542073 |
| 1709.RS34 | Dermatophagoides farinae | Pyroglyphidae | - | PAKISTAN | BMOC 12-0723-002 | - | - | - | - | - | MG766248 | KJ542074 |
| 1709.RS36 | Dermatophagoides farinae | Pyroglyphidae | - | PAKISTAN | BMOC 12-0723-002 | - | - | - | - | - | MG766249 | KJ542076 |
| 1709.RS37 | Dermatophagoides farinae | Pyroglyphidae | - | PAKISTAN | BMOC 12-0723-002 | - | - | - | - | - | MG766250 | KJ542077 |
| 1709.RS40 | Dermatophagoides farinae | Pyroglyphidae | - | PAKISTAN | BMOC 12-0723-002 | - | - | - | - | - | MG766251 | KJ542078 |
| 1709.RS14 | Dermatophagoides farinae | Pyroglyphidae | - | PAKISTAN | BMOC 12-0723-002 | - | - | - | - | - | - | - |
| 1709.RS25 | Dermatophagoides farinae | Pyroglyphidae | - | PAKISTAN | BMOC 12-0723-002 | - | - | - | - | - | - | KJ542067 |
| 1709.RS29 | Dermatophagoides farinae | Pyroglyphidae | - | PAKISTAN | BMOC 12-0723-002 | - | - | - | - | - | - | KJ542070 |
| 1709.RS41 | Dermatophagoides farinae | Pyroglyphidae | - | PAKISTAN | BMOC 12-0723-002 | - | - | - | - | - | - | KJ542079 |
| 1709.RS31 | Dermatophagoides farinae | Pyroglyphidae | - | PAKISTAN | BMOC 12-0723-002 | - | - | - | - | - | - | KJ542072 |
| 1709.RS35 | Dermatophagoides farinae | Pyroglyphidae | - | PAKISTAN | BMOC 12-0723-002 | - | - | - | - | - | - | KJ542075 |
| 1475.RS06 | Dermatophagoides pteronyssinus | Pyroglyphidae | - | USA | BMOC 12-0723-001 | - | - | - | - | - | MG766252 | KJ542086 |
| 1475.RS45 | Dermatophagoides pteronyssinus | Pyroglyphidae | - | USA | BMOC 12-0723-001 | - | - | - | - | - | MG766253 | KJ542092 |
| 1475.RS51 | Dermatophagoides pteronyssinus | Pyroglyphidae | - | USA | BMOC 12-0723-001 | - | - | - | - | - | MG766254 | KJ542093 |
| 1475.RS52 | Dermatophagoides pteronyssinus | Pyroglyphidae | - | USA | BMOC 12-0723-001 | - | - | - | - | - | MG766255 | KJ542094 |
| 1710.RS18 | Dermatophagoides pteronyssinus | Pyroglyphidae | - | PAKISTAN | BMOC 12-0723-002 | - | - | - | - | - | MG766256 | KJ542089 |
| 1710.RS19 | Dermatophagoides pteronyssinus | Pyroglyphidae | - | PAKISTAN | BMOC 12-0723-002 | - | - | - | - | - | MG766257 | KJ542090 |
| 1710.RS24 | Dermatophagoides pteronyssinus | Pyroglyphidae | - | PAKISTAN | BMOC 12-0723-002 | - | - | - | - | - | MG766258 | KJ542091 |
| 1710.RS65 | Dermatophagoides pteronyssinus | Pyroglyphidae | - | PAKISTAN | BMOC 12-0723-002 | - | - | - | - | - | MG766259 | KJ542095 |
| 1710.RS12 | Dermatophagoides pteronyssinus | Pyroglyphidae | - | PAKISTAN | BMOC 12-0723-002 | - | - | - | - | - | - | KJ542087 |
| 1710.RS16 | Dermatophagoides pteronyssinus | Pyroglyphidae | - | PAKISTAN | BMOC 12-0723-002 | - | - | - | - | - | - | KJ542088 |
| 1710.RS68 | Dermatophagoides pteronyssinus | Pyroglyphidae | - | PAKISTAN | BMOC 12-0723-002 | - | - | - | - | - | - | KJ542096 |
| 1710.RS73 | Dermatophagoides pteronyssinus | Pyroglyphidae | - | PAKISTAN | BMOC 12-0723-002 | - | - | - | - | - | - | KJ542097 |
| - | Dermatophagoides pteronyssinus | Pyroglyphidae | - | IRELAND | - | - | - | - | - | - | - | MQNO02000003 |
| - | Dermatophagoides farinae | Pyroglyphidae | - | SINGAPORE | - | - | - | - | - | - | - | ASGP01004202 |

*Gregarine sequence amplified, GenBank accession number MG766260.
