# Supplementary material for: Cox1 barcoding versus multilocus species delimitation: validation of two mite species with contrasting effective population sizes
Source: Parasit Vectors. 2019 Jan 5;12:8. doi: 10.1186/s13071-018-3242-5 (PMC6321676; doi:10.1186/s13071-018-3242-5)

Maximum likelihood CO1 tree of three species of house dust mites (*Dermatophagoides*) showing positions of low quality GenBank sequences

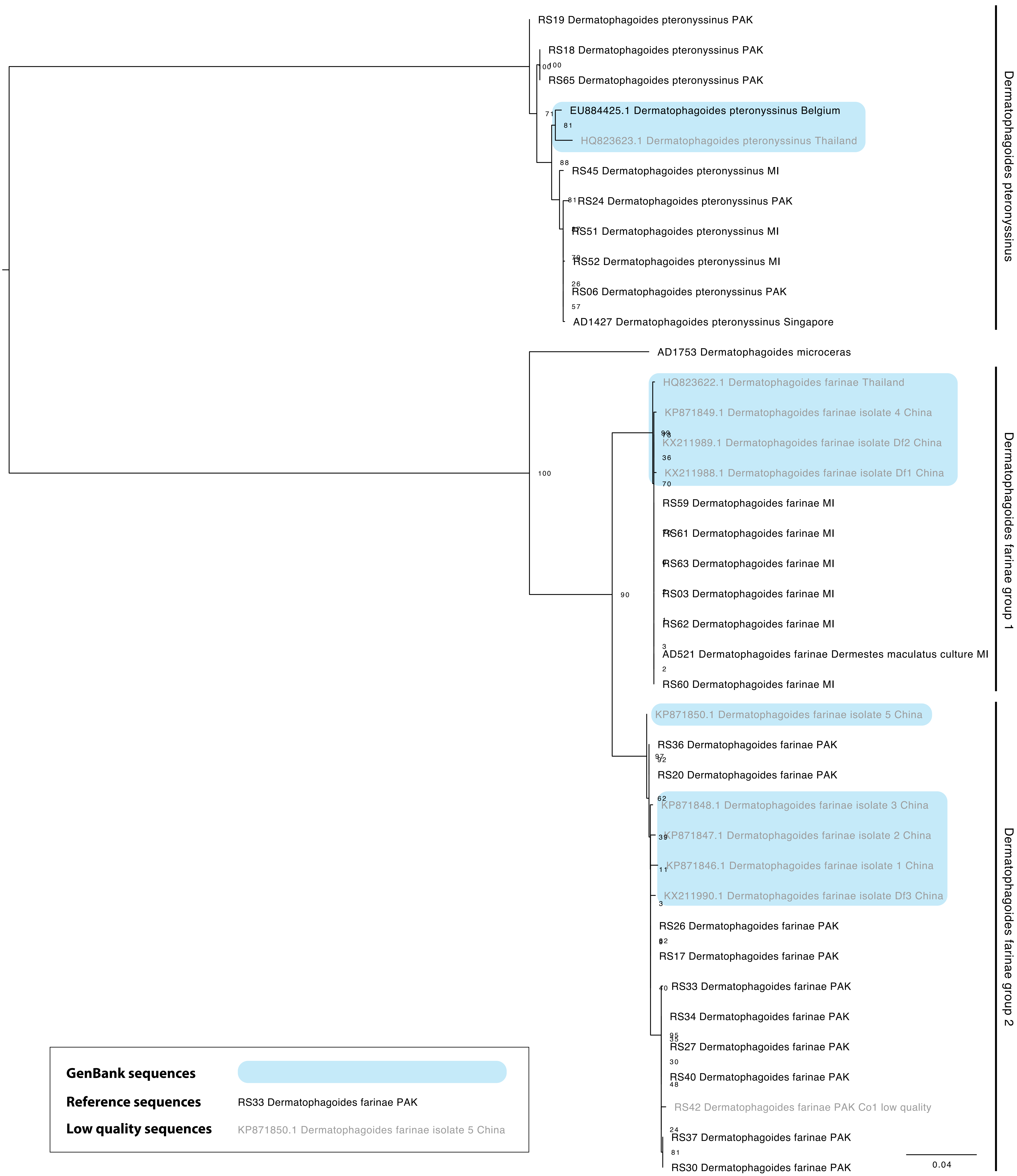

Supplement: Supplementary file 3 — Figure S1. Position of low quality sequences of two species of Dermatophagoides on phylogenetic tree. Low quality sequences are identified in nexus file S4 (amino acid color-coded alignment should be viewed in the program Mesquite). (PDF 164 kb) [file 13071_2018_3242_MOESM3_ESM.pdf]
