# Supplementary material for: Cox1 barcoding versus multilocus species delimitation: validation of two mite species with contrasting effective population sizes
Source: Parasit Vectors. 2019 Jan 5;12:8. doi: 10.1186/s13071-018-3242-5 (PMC6321676; doi:10.1186/s13071-018-3242-5)
Supplement: Supplementary file 5 — Table S2. Within- and among-species genetic distances of 7 loci of 10 putative species. (DOCX 79 kb) [file 13071_2018_3242_MOESM5_ESM.docx]

**Additional file 5: Table S2**. Within- and among-species genetic distances of 7 loci of 10 putative species. Nucleotide data are presented as K2P distances, while amino acid data are given as uncorrected distances, multiplied by 3. Putative species are annotated as having / not having clear morphological boundaries and whether they are host specific.

|  | **Species** | **Morphological**  **differences** | **Host specificity** | **Distance type** | **Sister species** |  | **Nucleotide K2P distance,%** | | **Amino acid distance,%** | |
| --- | --- | --- | --- | --- | --- | --- | --- | --- | --- | --- |
|  |  |  |  |  |  | **Locus** | **min** | **max** | **min** | **max** |
| 1 | Dermatophagoides farinae | clear | free-living | within species | microceras | CO1 | 0.0000 | 4.1890 | 0.0000 | 0.1618 |
| 2 | Dermatophagoides pteronyssinus | clear | free-living | within species | evansi | CO1 | 0.0000 | 1.9703 | 0.0000 | 0.0809 |
| 3 | Chorioptes bovis | clear | specific | within species | sweatmani | CO1 | na | na | na | na |
| 4 | Onychalges sp2 ex Lagonosticta rhodopareia | clear | specific | within species | pachyspathus ex Estrilda astrild | CO1 | na | na | na | na |
| 5 | Picalgoides sp n ex Colaptes auratus | clear | specific | within species | aff pteroglossorum ex Ramphastos sulfuratus | CO1 | na | na | na | na |
| 6 | Gymnoglyphus longior | weak | free-living | within species | osu | CO1 | na | na | na | na |
| 7 | Caparinia tripilis | weak | specific | within species | ictonyctis | CO1 | 0.0000 | 1.2240 | 0.0000 | 0.0000 |
| 8 | Psoroptes ovis | clear | not specific | within species | NA | CO1 | 0.0000 | 6.0061 | 0.0000 | 1.4599 |
| 9 | Psoroptes ovis ex Oryctolagus cuniculus | no | specific | within species | ex Ovis aries | CO1 | 0.0000 | 1.2938 | 0.0000 | 0.1667 |
| 10 | gen aff Paralgopsis ex Colaptes auratus | no | specific | within species | ex Melanerpes aurifrons | CO1 | na | na | na | na |
| 1 | Dermatophagoides farinae | clear | free-living | with sister species | microceras | CO1 | 9.3369 | 10.0249 | 0.2427 | 0.3236 |
| 2 | Dermatophagoides pteronyssinus | clear | free-living | with sister species | evansi | CO1 | 14.9844 | 15.4977 | 0.4045 | 0.4854 |
| 3 | Chorioptes bovis | clear | specific | with sister species | sweatmani | CO1 | 11.3534 | 11.3534 | 0.6472 | 0.6472 |
| 4 | Onychalges sp2 ex Lagonosticta rhodopareia | clear | specific | with sister species | pachyspathus ex Estrilda astrild | CO1 | 13.6796 | 13.6796 | 0.4045 | 0.4045 |
| 5 | Picalgoides sp n ex Colaptes auratus | clear | specific | with sister species | aff pteroglossorum ex Ramphastos sulfuratus | CO1 | 13.4594 | 13.4594 | 1.0518 | 1.0518 |
| 6 | Gymnoglyphus longior | weak | free-living | with sister species | osu | CO1 | 9.5194 | 9.5194 | 0.7317 | 0.7317 |
| 7 | Caparinia tripilis | weak | specific | with sister species | ictonyctis | CO1 | 7.4815 | 7.7652 | 0.3236 | 0.3236 |
| 8 | Psoroptes ovis | clear | not specific | with sister species | NA | CO1 | na | na | na | na |
| 9 | Psoroptes ovis ex Oryctolagus cuniculus | no | specific | with sister species | ex Ovis aries | CO1 | 4.2953 | 6.0061 | 0.8333 | 1.4599 |
| 10 | gen aff Paralgopsis ex Colaptes auratus | no | specific | with sister species | ex Melanerpes aurifrons | CO1 | 6.9520 | 6.9520 | 0.4045 | 0.4045 |
| 1 | Dermatophagoides farinae | clear | free-living | with sister species | microceras | 18S | 0.8691 | 0.8691 | na | na |
| 2 | Dermatophagoides pteronyssinus | clear | free-living | with sister species | evansi | 18S | 0.5192 | 0.5192 | na | na |
| 3 | Chorioptes bovis | clear | specific | with sister species | sweatmani | 18S | 0.9258 | 0.9641 | na | na |
| 4 | Onychalges sp2 ex Lagonosticta rhodopareia | clear | specific | with sister species | pachyspathus ex Estrilda astrild | 18S | 0.4043 | 0.4043 | na | na |
| 5 | Picalgoides sp n ex Colaptes auratus | clear | specific | with sister species | aff pteroglossorum ex Ramphastos sulfuratus | 18S | 0.9267 | 0.9267 | na | na |
| 6 | Gymnoglyphus longior | weak | free-living | with sister species | osu | 18S | 0.0000 | 0.0000 | na | na |
| 7 | Caparinia tripilis | weak | specific | with sister species | ictonyctis | 18S | na | na | na | na |
| 8 | Psoroptes ovis | clear | not specific | with sister species | NA | 18S | na | na | na | na |
| 9 | Psoroptes ovis ex Oryctolagus cuniculus | no | specific | with sister species | ex Ovis aries | 18S | na | na | na | na |
| 10 | gen aff Paralgopsis ex Colaptes auratus | no | specific | with sister species | ex Melanerpes aurifrons | 18S | 0.0000 | 0.0000 | na | na |
| 1 | Dermatophagoides farinae | clear | free-living | with sister species | microceras | 28S | 1.6884 | 1.6884 | na | na |
| 2 | Dermatophagoides pteronyssinus | clear | free-living | with sister species | evansi | 28S | 1.3142 | 1.3142 | na | na |
| 3 | Chorioptes bovis | clear | specific | with sister species | sweatmani | 28S | 3.6173 | 3.6173 | na | na |
| 4 | Onychalges sp2 ex Lagonosticta rhodopareia | clear | specific | with sister species | pachyspathus ex Estrilda astrild | 28S | 1.2775 | 1.2775 | na | na |
| 5 | Picalgoides sp n ex Colaptes auratus | clear | specific | with sister species | aff pteroglossorum ex Ramphastos sulfuratus | 28S | 2.5787 | 2.5787 | na | na |
| 6 | Gymnoglyphus longior | weak | free-living | with sister species | osu | 28S | 0.3302 | 0.3302 | na | na |
| 7 | Caparinia tripilis | weak | specific | with sister species | ictonyctis | 28S | 0.0632 | 0.0632 | na | na |
| 8 | Psoroptes ovis | clear | not specific | with sister species | NA | 28S | na | na | na | na |
| 9 | Psoroptes ovis ex Oryctolagus cuniculus | no | specific | with sister species | ex Ovis aries | 28S | na | na | na | na |
| 10 | gen aff Paralgopsis ex Colaptes auratus | no | specific | with sister species | ex Melanerpes aurifrons | 28S | 0.2196 | 0.2196 | na | na |
| 1 | Dermatophagoides farinae | clear | free-living | within species | microceras | CPW2 | 0.0000 | 0.9451 | 0.0000 | 0.1065 |
| 2 | Dermatophagoides pteronyssinus | clear | free-living | within species | evansi | CPW2 | 0.0000 | 0.4824 | 0.0000 | 0.4193 |
| 3 | Chorioptes bovis | clear | specific | within species | sweatmani | CPW2 | na | na | na | na |
| 4 | Onychalges sp2 ex Lagonosticta rhodopareia | clear | specific | within species | pachyspathus ex Estrilda astrild | CPW2 | na | na | na | na |
| 5 | Picalgoides sp n ex Colaptes auratus | clear | specific | within species | aff pteroglossorum ex Ramphastos sulfuratus | CPW2 | na | na | na | na |
| 6 | Gymnoglyphus longior | weak | free-living | within species | osu | CPW2 | na | na | na | na |
| 7 | Caparinia tripilis | weak | specific | within species | ictonyctis | CPW2 | na | na | na | na |
| 8 | Psoroptes ovis | clear | not specific | within species | NA | CPW2 | na | na | na | na |
| 9 | Psoroptes ovis ex Oryctolagus cuniculus | no | specific | within species | ex Ovis aries | CPW2 | na | na | na | na |
| 10 | gen aff Paralgopsis ex Colaptes auratus | no | specific | within species | ex Melanerpes aurifrons | CPW2 | na | na | na | na |
| 1 | Dermatophagoides farinae | clear | free-living | with sister species | microceras | EF1-α | 0.7607 | 0.7607 | 0.2841 | 0.2841 |
| 2 | Dermatophagoides pteronyssinus | clear | free-living | with sister species | evansi | EF1-α | 2.5070 | 2.5070 | 0.2841 | 0.2841 |
| 3 | Chorioptes bovis | clear | specific | with sister species | sweatmani | EF1-α | 3.2146 | 3.2146 | 0.0000 | 0.0000 |
| 4 | Onychalges sp2 ex Lagonosticta rhodopareia | clear | specific | with sister species | pachyspathus ex Estrilda astrild | EF1-α | 1.5308 | 1.5308 | 0.0000 | 0.0000 |
| 5 | Picalgoides sp n ex Colaptes auratus | clear | specific | with sister species | aff pteroglossorum ex Ramphastos sulfuratus | EF1-α | 6.0463 | 6.0463 | 0.2841 | 0.2841 |
| 6 | Gymnoglyphus longior | weak | free-living | with sister species | osu | EF1-α | 0.8577 | 0.8577 | 0.0000 | 0.0000 |
| 7 | Caparinia tripilis | weak | specific | with sister species | ictonyctis | EF1-α | 0.0946 | 0.0946 | 0.0000 | 0.0000 |
| 8 | Psoroptes ovis | clear | not specific | with sister species | NA | EF1-α | na | na | na | na |
| 9 | Psoroptes ovis ex Oryctolagus cuniculus | no | specific | with sister species | ex Ovis aries | EF1-α | na | na | na | na |
| 10 | gen aff Paralgopsis ex Colaptes auratus | no | specific | with sister species | ex Melanerpes aurifrons | EF1-α | 0.0000 | 0.0000 | 0.0000 | 0.0000 |
| 1 | Dermatophagoides farinae | clear | free-living | with sister species | microceras | SRP54 | 1.7246 | 1.7246 | 0.4454 | 0.4454 |
| 2 | Dermatophagoides pteronyssinus | clear | free-living | with sister species | evansi | SRP54 | 3.4240 | 3.4240 | 0.0000 | 0.0000 |
| 3 | Chorioptes bovis | clear | specific | with sister species | sweatmani | SRP54 | 5.6594 | 5.6594 | 0.0000 | 0.0000 |
| 4 | Onychalges sp2 ex Lagonosticta rhodopareia | clear | specific | with sister species | pachyspathus ex Estrilda astrild | SRP54 | 5.6305 | 5.6305 | 0.2227 | 0.2227 |
| 5 | Picalgoides sp n ex Colaptes auratus | clear | specific | with sister species | aff pteroglossorum ex Ramphastos sulfuratus | SRP54 | 8.0358 | 8.0358 | 0.2970 | 0.2970 |
| 6 | Gymnoglyphus longior | weak | free-living | with sister species | osu | SRP54 | 1.1215 | 1.1215 | 0.0000 | 0.0000 |
| 7 | Caparinia tripilis | weak | specific | with sister species | ictonyctis | SRP54 | 0.2972 | 0.2972 | 0.0000 | 0.0000 |
| 8 | Psoroptes ovis | clear | not specific | with sister species | NA | SRP54 | na | na | na | na |
| 9 | Psoroptes ovis ex Oryctolagus cuniculus | no | specific | with sister species | ex Ovis aries | SRP54 | na | na | na | na |
| 10 | gen aff Paralgopsis ex Colaptes auratus | no | specific | with sister species | ex Melanerpes aurifrons | SRP54 | 0.2226 | 0.2226 | 0.0000 | 0.0000 |
| 1 | Dermatophagoides farinae | clear | free-living | with sister species | microceras | HSP70 | 1.9088 | 1.9088 | 0.4695 | 0.4695 |
| 2 | Dermatophagoides pteronyssinus | clear | free-living | with sister species | evansi | HSP70 | 5.6992 | 5.6992 | 0.2347 | 0.2347 |
| 3 | Chorioptes bovis | clear | specific | with sister species | sweatmani | HSP70 | 3.8718 | 3.8718 | 0.0587 | 0.0587 |
| 4 | Onychalges sp2 ex Lagonosticta rhodopareia | clear | specific | with sister species | pachyspathus ex Estrilda astrild | HSP70 | 4.7331 | 4.7331 | 0.1761 | 0.1761 |
| 5 | Picalgoides sp n ex Colaptes auratus | clear | specific | with sister species | aff pteroglossorum ex Ramphastos sulfuratus | HSP70 | 7.8612 | 7.8612 | 0.4108 | 0.4108 |
| 6 | Gymnoglyphus longior | weak | free-living | with sister species | osu | HSP70 | 0.4716 | 0.4716 | 0.0587 | 0.0587 |
| 7 | Caparinia tripilis | weak | specific | with sister species | ictonyctis | HSP70 | 0.5301 | 0.5301 | 0.0587 | 0.0587 |
| 8 | Psoroptes ovis | clear | not specific | with sister species | NA | HSP70 | na | na | na | na |
| 9 | Psoroptes ovis ex Oryctolagus cuniculus | no | specific | with sister species | ex Ovis aries | HSP70 | na | na | na | na |
| 10 | gen aff Paralgopsis ex Colaptes auratus | no | specific | with sister species | ex Melanerpes aurifrons | HSP70 | 0.2352 | 0.2352 | 0.0587 | 0.0587 |
