# Supplementary figures and images for: Cox1 barcoding versus multilocus species delimitation: validation of two mite species with contrasting effective population sizes
Source: Parasit Vectors. 2019 Jan 5;12:8. doi: 10.1186/s13071-018-3242-5 (PMC6321676; doi:10.1186/s13071-018-3242-5)

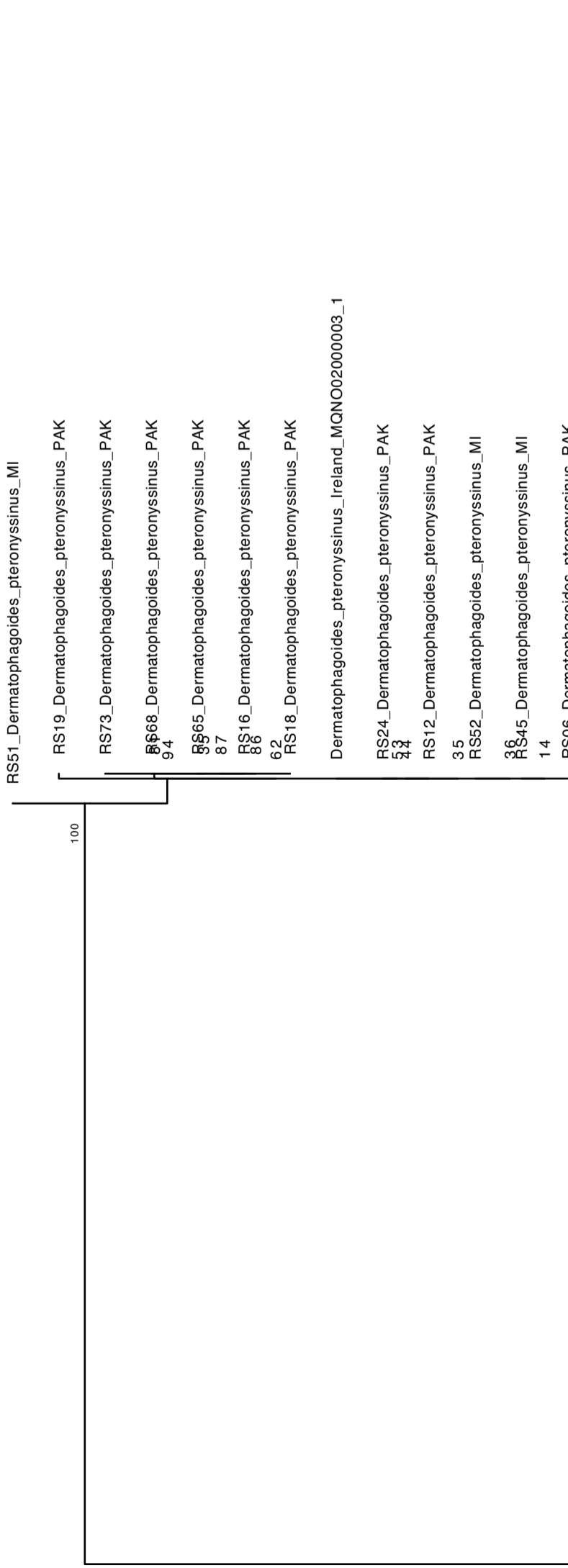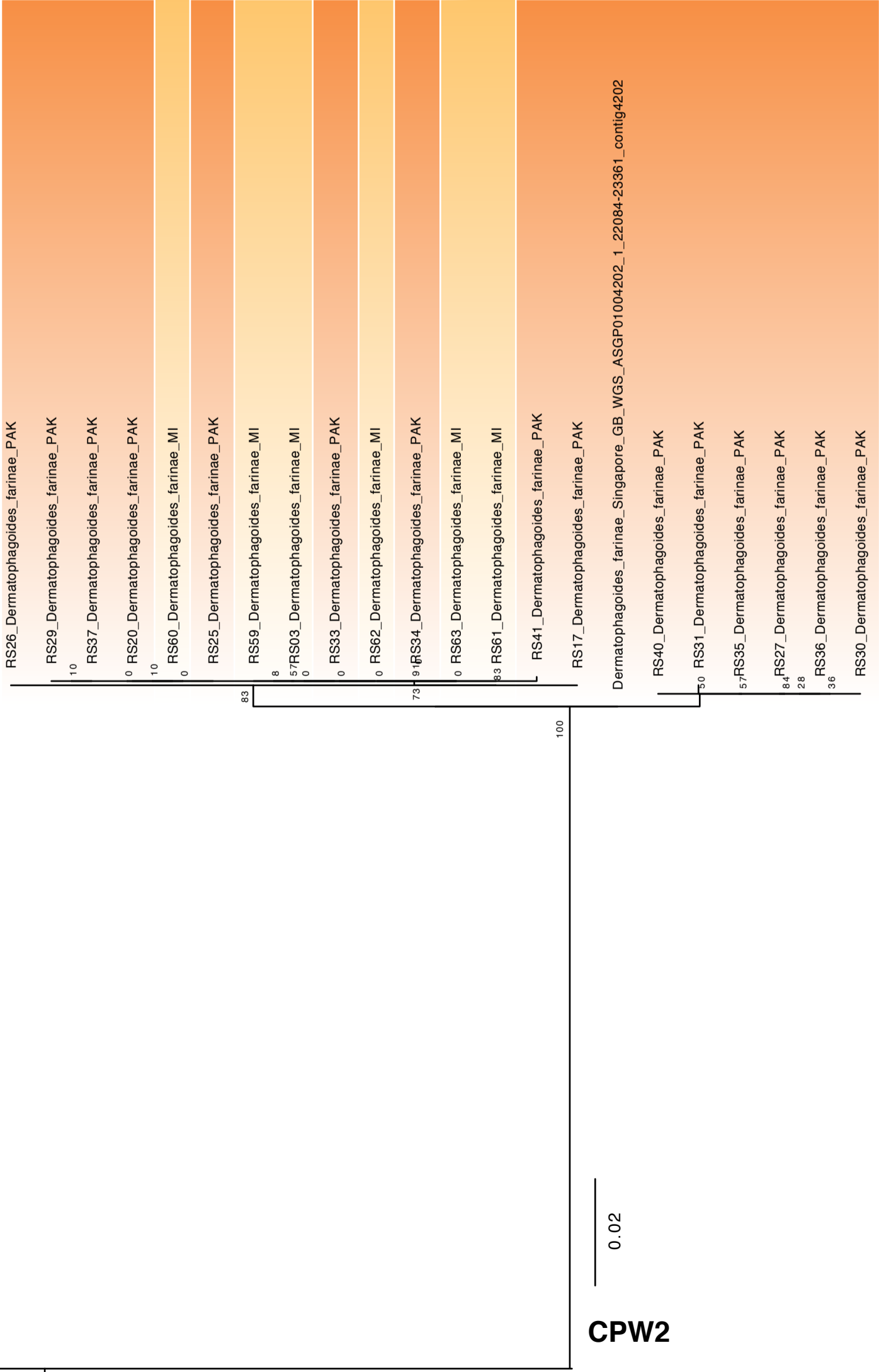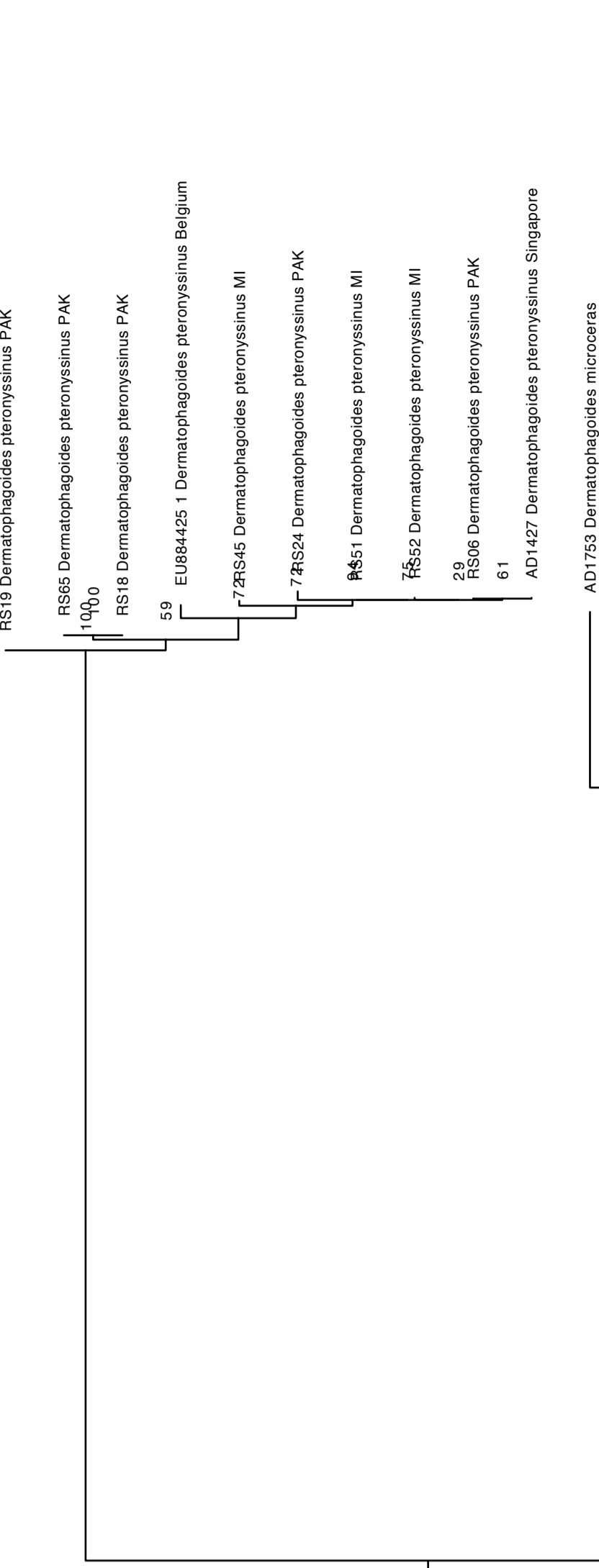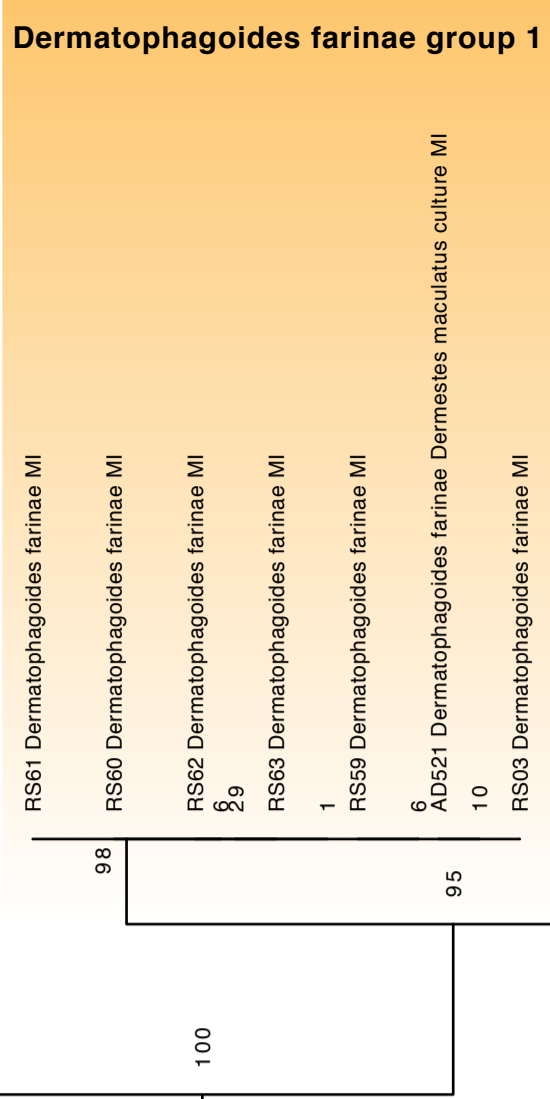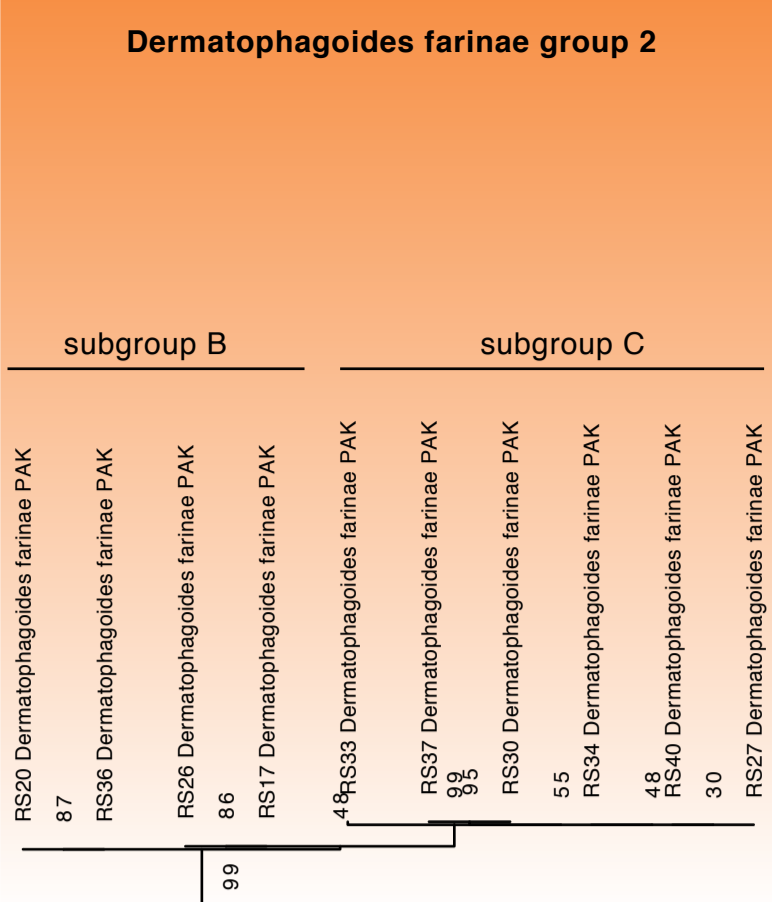

0.08

C01

0.02

CPW2

Supplement: Supplementary file 6 — Figure S2. Dermatophagoides farinae CO1 and CPW2 gene trees inferred in a Maximum Likelihood framework (RAxML). Important groupings are indicated. (PDF 215 kb) [file 13071_2018_3242_MOESM6_ESM.pdf]

Dermatophagoides farinae (DF)

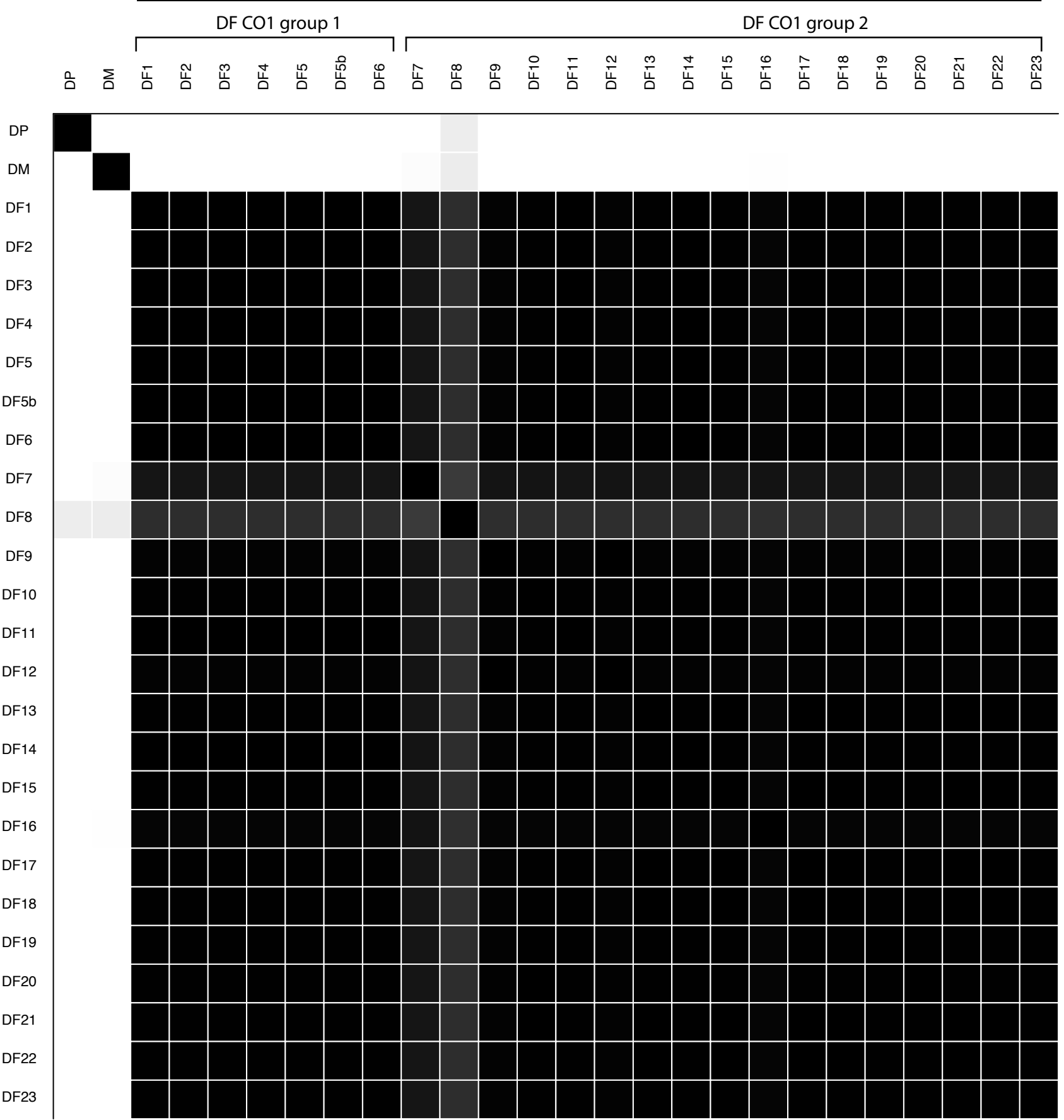

STACEY minimal clusters

Supplement: Supplementary file 8 — Figure S3. Similarity matrix of STACEY species discovery analysis of the Dermatophagoides dataset. (PDF 138 kb) [file 13071_2018_3242_MOESM8_ESM.pdf]
